# Thiazoles with cyclopropyl fragment as antifungal, anticonvulsant, and anti-*Toxoplasma gondii* agents: synthesis, toxicity evaluation, and molecular docking study

**DOI:** 10.1007/s00044-018-2221-x

**Published:** 2018-07-21

**Authors:** Krzysztof Z. Łączkowski, Natalia Konklewska, Anna Biernasiuk, Anna Malm, Kinga Sałat, Anna Furgała, Katarzyna Dzitko, Adrian Bekier, Angelika Baranowska-Łączkowska, Agata Paneth

**Affiliations:** 1 Department of Chemical Technology and Pharmaceuticals, Faculty of Pharmacy, Collegium Medicum, Nicolaus Copernicus University, Jurasza 2, 85-089 Bydgoszcz, Poland; 2 Department of Pharmaceutical Microbiology, Faculty of Pharmacy, Medical University, Chodźki 1, 20-093 Lublin, Poland; 3 Chair of Pharmacodynamics, Faculty of Pharmacy, Jagiellonian University, Medical College, Medyczna 9, 30-688 Krakow, Poland; 4 Department of Immunoparasitology, Faculty of Biology and Environmental Protection, University of Lodz, Banacha 12/16, 90-237 Lodz, Poland; 5 Institute of Physics, Kazimierz Wielki University, Plac Weyssenhoffa 11, 85-072 Bydgoszcz, Poland; 6 Department of Organic Chemistry, Faculty of Pharmacy, Medical University of Lublin, Chodźki 4a, 20-093 Lublin, Poland

**Keywords:** Cyclopropanecarboxaldehyde, Thiazole, *Candida* spp., Anticonvulsant activity, *Toxoplasma gondii*

## Abstract

Synthesis and investigation of antifungal, anticonvulsant and anti-*Toxoplasma gondii* activities of ten novel (2-(cyclopropylmethylidene)hydrazinyl)thiazole **3a**–**3j** are presented. Among the derivatives, compounds **3a**–**3d** and **3f**–**3j** possess very high activity against *Candida* spp. ATCC with MIC = 0.015–7.81 µg/ml. Compounds **3a**–**3d** and **3f**–**3j** possess also very high activity towards most of strains of *Candida* spp. isolated from clinical materials with MIC = 0.015–7.81 µg/ml. The activity of these compounds is similar and even higher than the activity of nystatin used as positive control. Additionally, compounds **3c** and **3e** showed interesting anticonvulsant activities in the MES test, whereas compounds **3f** and **3i** demonstrated the anticonvulsant activity in PTZ-induced seizures. Noteworthy, none of these compounds impaired animals’ motor skills in the rotarod test. Moreover, thiazoles **3a**, **3h**, and **3j** showed significant anti-*Toxoplasma gondii* activity, with IC_50_ values 31–52 times lower than those observed for sulfadiazine. The results of the cytotoxicity evaluation, *anti-Candida* spp. and anti-*Toxoplasma gondii* activity studies showed that *Candida* spp. and *Toxoplasma gondii* growth was inhibited at non-cytotoxic concentrations for the mouse L929 fibroblast and the African green monkey kidney (VERO) cells. Molecular docking studies indicated secreted aspartic proteinase (SAP) as possible antifungal target.

## Introduction

*Candida* spp. is the most common group of nosocomial pathogens that cause invasive fungal infection leading to hospitalizations and death (Richards et al. 2000). The majority of candidiasis cases are caused by *Candida albicans*, however non-*C. albicans*, such as *Candida glabrata*, *Candida parapsilosis*, *Candida tropicalis* and *Candida krusei* has recently been found an important group of pathogens that also cause bloodstream infections (IC) (Macphail et al. 2002). The ratio of individual pathogens is variable and depends on many factors, such as the age of the patient, geography, underlying diseases and the type of recently used drugs (Wu et al. 2014). *C. glabrata* is the second most commonly isolated pathogen, occurring especially in the elderly people suffering from cancer and under azole prophylaxis, while *C. parapsilosis* occurs mainly in catheterized neonates in southern Europe, Asia and South America. In contrast, *C. krusei* is a particularly common pathogen in people undergoing corticosteroid therapy and with hematologic malignancies (Guinea 2014).

There are many groups of drugs used to fight candidemia, however the irresponsible and widespread use of antifungals has been accompanied by multi-drug resistance of clinical isolates of *Candida* spp. (Nucci and Marr 2005). In 2016, the Infectious Disease Society of America (IDSA) published new guidelines for the use of echinocandins, azoles and also lipid formulations of amphotericin B in the treatment of candidemia, and other forms of invasive candidiasis (Pappas et al. 2016).

Epilepsy is a common neurological disorder that affects millions of people worldwide. This disease is featured by a strong propensity towards unprovoked seizures which are caused by various structural or functional impairments within the brain. Available anti-epileptic drugs are regarded to be symptomatic treatments being unable to cure epilepsy or prevent its development and they fail to control epileptic activity in about 30% of patients. Moreover, many of these drugs have serious adverse effects that result in poor tolerability and a reduced quality of patient’s life, resulting in a strong medical demand for novel treatment strategies for epilepsy (Lamberink et al. 2017).

Infections of the central nervous system (CNS) are regarded as one of major risk factors for seizure onset. So called “acquired epilepsy” might occur at any age of an individual’s life and this type of epilepsy might be considered as an under-recognized long-term complication of infections within the CNS (Vezzani et al. 2016). It has been shown that not only bacterial (typical bacterial meningitis, tuberculosis), viral (HSV, HHV-6), parasitic (cerebral toxoplasmosis, neurocysticercosis, and malaria) but also fungal neuroinfections caused by Candida, Cryptococcus, Coccidioides, Aspergillus, Blastomyces, and Histoplasma may induce seizures (Sander and Perucca 2003; Bittencourt et al. 1999) that may occur at any stage of fungal infections (Vezzani et al. 2016).

Taking into consideration a strong link between seizures and microbial infections, in addition to search for drugs that have high antifungal activity, we also decided to continue the search of “hybrid” drug candidates which possess antifungal, anticonvulsant, and anti-toxoplasmosis properties. Such compounds seem to be particularly interesting in terms of their potential development and application in the treatment of microbial infection-related seizures.

Hydrazinylthiazole moiety seems to be a good scaffold to generate a variety of antibacterial (Karegoudar et al. 2008; Mohammad et al. 2015; Zhao et al. 2017; Łączkowski et al. 2016a, 2017), antifungal (Łączkowski et al. 2016b; Carradori et al. 2013; Chimenti et al. 2007), anti-*Trypanosoma cruzi* (de Oliveira Filho et al. 2017), and anticancer (Łączkowski et al. 2016c; Łączkowski et al. 2014a) drugs. Our earlier studies have shown that incorporation of cyclopentylmethylene and tetrahydro-2*H*-thiopyran-4-yl fragments with hydrazinylthiazole moiety was a successful strategy to receive significant anticonvulsant activity in mouse models of seizures (Łączkowski et al. 2016d) and high antifungal activity against clinical isolates of *Candida* spp. (Łączkowski et al. 2016a).

Recently, more drugs containing a cyclopropyl group have reached to the phase of clinical trials. In addition, eight of the 200 best-selling FDA-approved drugs contain a cyclopropane ring. A lot of interest in this ring is due to its unique properties, such as planarity of the cyclopropane ring, the C–C bonds of character intermediate between *σ* and *π* and sometimes called “banana bonds”, as well as the C–H bonds shorter and stronger relative to C–H bonds in alkanes. These unique properties of the cyclopropane system make it an interesting substituent in newly designed drugs, that may result in increased activity, metabolic stability, bioavailability, brain permeability, and decrease in lipophilicity, i.e., ability to penetrate through various biological barriers (Talele 2016).

Considering the above facts we decided to design and synthesize ten novel hydrazinylthiazole derivatives containing cyclopropyl fragment and investigate their antifungal activity against a panel of reference strains of nineteen microorganisms from American Type Culture Collection (ATCC), routinely used for the evaluation of antimicrobials, and from clinical materials. In the in vivo part of this study anticonvulsant activity of ten novel thiazole derivatives were assessed in mouse models of chemically-induced and electrically-induced seizures. The four most active compounds at the highest anticonvulsant active dose was additionally tested for their influence on animals’ motor coordination. We also investigated intensity of *Toxoplasma gondii* RH virulent strain intracellular proliferation in the VERO host cells.

## Experimental

### Materials and methods

All experiments were carried out under air atmosphere unless stated otherwise. Reagents were generally the best quality commercial-grade products and were used without further purification. ^1^H nuclear magnetic resonance (NMR) (400 MHz) and ^13^C NMR (100 MHz) spectra were recorded on a Bruker Avance III multinuclear instrument. GC-EI-MS was performed by the Laboratory for Analysis of Organic Compounds and Polymers of the Centre for Molecular and Macromolecular Studies of the Polish Academy of Science in Łódź. MS spectra were recorded on a Finnigan MAT 95 spectrometer. Melting points were determined in open glass capillaries and are uncorrected. Analytical TLC was performed using Macherey-Nagel Polygram Sil G/UV_254_ 0.2 mm plates. Cyclopropanecarboxaldehyde, thiosemicarbazide and appropriate bromoketones were commercial materials (Aldrich).

#### 2-(Cyclopropylmethylidene)hydrazinecarboxamide (**2**)

Thiosemicarbazide (1.82 g, 20.0 mmol) was added to a stirred solution of cyclopropanecarboxaldehyde (**1**) (1.40 g, 20.0 mmol) in absolute ethyl alcohol (30 ml) and then (1.0 ml) of acetic acid was added. The reaction mixture was stirred under reflux for 20 h. Next, the reaction mixture was added to water (50 ml) and neutralized with NaHCO_3_ solution. The product was extracted with dichloromethane (2 × 100 ml), the solvent was evaporated in vacuo, and the solid was washed with diethyl ether to afford the desired product to yield 2.19 g (77%); mp 124–127 °C; eluent: dichloromethane/methanol (95:5), *R*_f_ = 0.56. ^1^H NMR (DMSO-d_6_, 400 MHz); *δ* (ppm): 0.64–0.70 (m, 2H, CH_2_); 0.82–0.88 (m, 2H, CH_2_); 1.51–1.62 (m, 1H, CH); 7.00 (d, 1H, CH, *J* = 7.5 Hz); 7.44 (bs, 1H, NH_2_); 7.88 (bs, 1H, NH_2_); 10.98 (bs, 1H, NH). ^13^C NMR (DMSO-d_6_, 100 MHz): *δ* (ppm) 6.73 (2C_cyclopropyl_); 13.79 (C_cyclopropyl_); 150.89 (C=N); 177.43 (C=S). Anal. calcd. for C_5_H_9_N_3_S: C, 41.93; H, 6.33; N, 29.34. Found: C, 41.95; H, 6.30; N, 29.37.

#### (*E*)-2-(2-(Cyclopropylmethylene)hydrazinyl)-4-(4-fluorophenyl)thiazole (3a): typical procedure

Carbothioamide **2** (0.143 g, 1.0 mmol) was added to a stirred solution of 2-bromo-1-(4-fluorophenyl)ethanone (0.217 g, 1.0 mmol) in absolute ethyl alcohol (15 ml). The reaction mixture was stirred at room temperature for 20 h. Next, the reaction mixture was added to water (50 ml) and neutralized with NaHCO_3_ solution. The product was extracted with dichloromethane (2 × 100 ml), the solvent was evaporated in vacuo, and the product was purified on silica gel column chromatography (230–400 mesh) using (dichloromethane/methanol, 95:5, *R*_f_ = 0.83) to afford the desired product: 0.18 g, 69%; mp 59–61 °C. ^1^H NMR (DMSO-d_6_, 400 MHz); *δ* (ppm): 0.64–0.70 (m, 2H, CH_2_); 0.82–0.88 (m, 2H, CH_2_); 1.51–1.62 (m, 1H, CH); 6.89 (d, 1H, CH, *J* = 8.32 Hz); 7.19–7.23 (m, 3H, 3CH); 7.83–7.87 (m, 2H, 2CH); 11.52 (bs, 1H, NH). ^13^C NMR (DMSO-d_6_, 100 MHz): *δ* (ppm) 6.35 (2C_cyclopropyl_); 13.97 (C_cyclopropyl_); 102.92 (C_thiazole_); 115.84 (d, 2C_Ar_, *J*_C–F_ = 21 Hz); 127.90 (d, 2C_Ar_, *J*_C–F_ = 8 Hz); 131.86 (C); 149.46 (C_thiazole_); 149.72 (C=N); 160.74 (C–F); 169.00 (C–NH). GC-EI-MS (*m*/*z*, %): 261 [(M^+^), 35], 194 (80), 152 (100). Anal. calcd. for C_13_H_12_FN_3_S: C, 59.75; H, 4.63; N, 16.08. Found: C, 59.73; H, 4.64; N, 16.11.

#### (*E*)-4-(4-Bromophenyl)-2-(2-(cyclopropylmethylene)hydrazinyl)thiazole (**3b**)

2-Bromo-1-(4-bromophenyl)ethanone was reacted with **2**. Yield: 0.50 g, 78%, (dichloromethane, *R*_f_ = 0.49); mp 165–167 °C. ^1^H NMR (DMSO-d_6_, 400 MHz); *δ* (ppm): 0.64–0.70 (m, 2H, CH_2_); 0.82–0.88 (m, 2H, CH_2_); 1.51–1.62 (m, 1H, CH); 6.92 (d, 1H, CH, *J* = 7.7 Hz); 7.29 (s, 1H, CH); 7.58 (d, 2H, 2CH, *J* = 8.6 Hz); 7.76 (d, 2H, 2CH, *J* = 8.6 Hz); 11.56 (bs, 1H, NH). ^13^C NMR (DMSO-d_6_, 100 MHz): *δ* (ppm) 6.57 (2C_cyclopropyl_); 14.05 (C_cyclopropyl_); 104.41 (C_thiazole_); 121.34 (C); 128.16 (2C_Ar_); 132.03 (2C_Ar_); 133.20 (C); 147.56 (C_thiazole_); 151.59 (C=N); 168.99 (C–NH). GC-EI-MS (*m*/*z*, %): 321 [(M^+^), 30], 256 (60), 174 (100). Anal. calcd. for C_13_H_12_BrN_3_S: C, 48.46; H, 3.75; N, 13.04. Found: C, 48.44; H, 3.77; N, 13.08.

#### (*E*)-4-(4-Chlorophenyl)-2-(2-(cyclopropylmethylene)hydrazinyl)thiazole (**3c**)

2-Bromo-1-(4-chlorophenyl)ethanone was reacted with **2**. Yield: 0.29 g, 52%, (dichloromethane, *R*_f_ = 0.48); mp 162–163 °C. ^1^H NMR (DMSO-d_6_, 400 MHz); *δ* (ppm): 0.64–0.70 (m, 2H, CH_2_); 0.82–0.88 (m, 2H, CH_2_); 1.51–1.62 (m, 1H, CH); 6.89–6.99 (m, 1H, CH); 7.29 (s, 1H, CH); 7.45 (d, 2H, 2CH, *J* = 8.36 Hz); 7.82 (d, 2H, 2CH, *J* = 8.36 Hz); 11.63 (bs, 1H, NH). ^13^C NMR (DMSO-d_6_, 100 MHz): *δ* (ppm) 6.49 (2C_cyclopropyl_); 14.02 (C_cyclopropyl_); 104.22 (C_thiazole_); 127.78 (2C_Ar_); 129.08 (2C_Ar_); 132.52 (C); 133.32 (C); 148.30 (C_thiazole_); 150.83 (C=N); 169.02 (C–NH). GC-EI-MS (*m*/*z*, %): 277 [(M^+^), 45], 210 (100), 174 (55), 168 (65). Anal. calcd. for C_13_H_12_ClN_3_S: C, 56.21; H, 4.35; N, 15.13. Found: C, 56.20; H, 4.39; N, 15.16.

#### (*E*)-2-(2-(Cyclopropylmethylene)hydrazinyl)-4-*p*-tolylthiazole (**3d**)

2-Bromo-1-(4-methylphenyl)ethanone was reacted with **2**. Yield: 0.36 g, 70%, (dichloromethane, *R*_f_ = 0.24); mp 158–160 °C. ^1^H NMR (DMSO-d_6_, 400 MHz); *δ* (ppm): 0.64–0.70 (m, 2H, CH_2_); 0.82–0.88 (m, 2H, CH_2_); 1.51–1.62 (m, 1H, CH); 2.31 (s, 3H, CH_3_); 6.94 (d, 1H, CH, *J* = 7.76 Hz); 7.14 (s, 1H, CH); 7.20 (d, 2H, 2CH, *J* = 8.00 Hz); 7.69 (d, 2H, 2CH, *J* = 8.00 Hz); 11.47 (bs, 1H, NH). ^13^C NMR (DMSO-d_6_, 100 MHz): *δ* (ppm) 6.74 (2C_cyclopropyl_); 14.12 (C_cyclopropyl_); 21.28 (C); 103.01 (C_thiazole_); 126.24 (2C_Ar_); 129.74 (2C_Ar_); 130.03 (C); 138.26 (C); 146.71 (C_thiazole_); 153.45 (C=N); 168.87 (C–NH). GC-EI-MS (*m*/*z*, %): 257 [(M^+^), 55], 190 (100), 148 (90). Anal. calcd. for C_14_H_15_N_3_S: C, 65.34; H, 5.87; N, 16.33. Found: C, 65.36; H, 5.85; N, 16.35.

#### (*E*)-2-(2-(Cyclopropylmethylene)hydrazinyl)-4-(4-(trifluoromethyl)phenyl)thiazole (3e)

2-Bromo-1-(4-trifluoromethylphenyl)ethanone was reacted with **2**. Yield: 0.57 g, 92%, (dichloromethane, *R*_f_ = 0.46); mp 132–134 °C. ^1^H NMR (DMSO-d_6_, 400 MHz); *δ* (ppm): 0.64–0.70 (m, 2H, CH_2_); 0.82–0.88 (m, 2H, CH_2_); 1.51–1.62 (m, 1H, CH); 6.91 (d, 1H, CH, *J* = 8.0 Hz); 7.46 (s, 1H, CH); 7.74 (d, 2H, 2CH, *J* = 8.6 Hz); 8.03 (d, 2H, 2CH, *J* = 8.6 Hz); 11.62 (bs, 1H, NH). ^13^C NMR (DMSO-d_6_, 100 MHz): *δ* (ppm) 6.38 (2C_cyclopropyl_); 13.98 (C_cyclopropyl_); 105.97 (C_thiazole_); 123.46 (C); 125.99 (2C_Ar_); 126.45 (2C_Ar_); 127.91 (q, C–F, *J*_C–F_ = 32 Hz); 138.91 (C); 149.27 (C_thiazole_); 149.72 (C=N); 169.18 (C–NH). GC-EI-MS (*m*/*z*, %): 311 [(M^+^), 35], 244 (100), 202 (60). Anal. calcd. for C_14_H_12_F_3_N_3_S: C, 54.01; H, 3.89; N, 13.50. Found: C, 53.98; H, 3.87; N, 13.54.

#### (*E*)-2-(2-(Cyclopropylmethylene)hydrazinyl)-4-(4-methoxyphenyl)thiazole (3f)

2-Bromo-1-(4-methoxyphenyl)ethanone was reacted with **2**. Yield: 0.38 g, 70%, (dichloromethane, *R*_f_ = 0.21); mp 149–152 °C. ^1^H NMR (DMSO-d_6_, 400 MHz); *δ* (ppm): 0.64–0.70 (m, 2H, CH_2_); 0.82–0.88 (m, 2H, CH_2_); 1.51–1.62 (m, 1H, CH); 6.96 (d, 3H, 3CH, *J* = 8.9 Hz); 7.06 (s, 1H, CH); 7.73 (d, 2H, 2CH, *J* = 8.9 Hz); 11.56 (bs, 1H, NH). ^13^C NMR (DMSO-d_6_, 100 MHz): *δ* (ppm) 6.74 (2C_cyclopropyl_); 14.14 (C_cyclopropyl_); 55.70 (C); 101.84 (C_thiazole_); 114.56 (2C_Ar_); 125.42 (C); 127.78 (2C_Ar_); 146.43 (C_thiazole_); 153.46 (C=N); 159.80 (C–O); 168.81 (C–NH). GC-EI-MS (*m*/*z*, %): 273 [(M^+^), 80], 206 (85), 164 (100), 149 (45). Anal. calcd. for C_14_H_15_N_3_OS: C, 61.51; H, 5.53; N, 15.37. Found: C, 61.53; H, 5.50; N, 15.40.

#### (*E*)-2-(2-(Cyclopropylmethylene)hydrazinyl)-4-(2,4-difluorophenyl)thiazole (3g)

2-Bromo-1-(2′4′-difluorophenyl)ethanone was reacted with **2**. Yield: 0.31 g, 55%, (dichloromethane, R_f_ = 0.46); mp 97–100 °C. ^1^H NMR (DMSO-d_6_, 400 MHz); *δ* (ppm): 0.64–0.70 (m, 2H, CH_2_); 0.82–0.88 (m, 2H, CH_2_); 1.51–1.62 (m, 1H, CH); 6.92 (d, 1H, CH, *J* = 7.7 Hz); 7.08–7.18 (m, 2H, 2CH); 7.26–7.36 (m, 1H, CH); 7.95–8.02 (m, 1H, CH); 11.59 (bs, 1H, NH). ^13^C NMR (DMSO-d_6_, 100 MHz): *δ* (ppm) 6.38 (2C_cyclopropyl_); 13.97 (C_cyclopropyl_); 104.88 (t, C, *J* = 27.0 Hz); 107.30 (d, C, *J* = 30.0 Hz); 112.20 (d, C, *J*_C–F_ = 21.0 Hz); 119.61 (C); 130.82 (C); 143.60 (C); 149.67 (C); 159.52 (dd, C, *J*_1_ = 13.0 Hz, *J*_2_ = 167.0 Hz); 162.00 (dd, C, *J*_1_ = 14.0 Hz, *J*_2_ = 162.0 Hz); 168.31 (C–NH). GC-EI-MS (*m*/*z*, %): 279 [(M^+^), 35], 212 (85), 170 (100). Anal. calcd. for C_13_H_11_F_2_N_3_S: C, 55.90; H, 3.97; N, 15.04. Found: C, 55.88; H, 3.98; N, 15.07.

#### (*E*)-4-(2-(2-(Cyclopropylmethylene)hydrazinyl)thiazol-4-yl)benzonitrile (**3h**)

4-(Bromoacetyl)benzonitrile was reacted with **2**. Yield: 0.54 g, 99%, (dichloromethane, *R*_f_ = 0.24); mp 168–171 °C. ^1^H NMR (DMSO-d_6_, 400 MHz); *δ* (ppm): 0.64–0.70 (m, 2H, CH_2_); 0.82–0.88 (m, 2H, CH_2_); 1.51–1.62 (m, 1H, CH); 6.96 (d, 1H, CH, *J* = 7.8 Hz); 7.53 (s, 1H, CH); 7.84 (d, 2H, 2CH, *J* = 8.7 Hz); 7.98 (d, 1H, CH, *J* = 8.7 Hz); 11.49 (bs, 1H, NH). ^13^C NMR (DMSO-d_6_, 100 MHz): *δ* (ppm) 6.49 (2C_cyclopropyl_); 14.02 (C_cyclopropyl_); 107.21 (C_thiazole_); 110.02 (C); 119.45 (C); 126.66 (2C_Ar_); 133.11 (2C_Ar_); 138.82 (C); 148.28 (C_thiazole_); 150.67 (C=N); 169.18 (C–NH). GC-EI-MS (*m*/*z*, %): 268 [(M^+^), 35], 201 (100), 159 (80). Anal. calcd. for C_14_H_12_N_4_S: C, 62.66; H, 4.51; N, 20.88. Found: C, 62.66; H, 4.50; N, 20.92.

#### (*E*)-4-(4-Azidophenyl)-2-(2-(cyclopropylmethylene)hydrazinyl)thiazole (**3i**)

2-Bromo-1-(4-azidophenyl)ethanone was reacted with **2**. Yield: 0.14 g, 83%, (dichloromethane, *R*_f_ = 0.31); mp 124–126 °C. ^1^H NMR (DMSO-d_6_, 400 MHz); *δ* (ppm): 0.64–0.70 (m, 2H, CH_2_); 0.82–0.88 (m, 2H, CH_2_); 1.51–1.62 (m, 1H, CH); 6.89 (d, 1H, CH, *J* = 7.44 Hz); 7.13 (d, 2H, 2CH, *J* = 8.92 Hz); 7.20 (s, 1H, CH); 7.85 (d, 2H, 2CH, *J* = 8.92 Hz); 11.54 (bs, 1H, NH). ^13^C NMR (DMSO-d_6_, 100 MHz): *δ* (ppm) 6.36 (2C_cyclopropyl_); 13.98 (C_cyclopropyl_); 103.07 (C_thiazole_); 119.74 (2C_Ar_); 127.55 (2C_Ar_); 132.36 (C); 138.64 (C); 149.45 (C_thiazole_); 149.96 (C=N); 168.95 (C–NH). GC-EI-MS (*m*/*z*, %): 284 [(M^+^), 30], 256 (100), 188 (65). Anal. calcd. for C_13_H_12_N_6_S: C, 54.91; H, 4.25; N, 29.56. Found: C, 54.94; H, 4.27; N, 29.61.

#### (*E*)-2-(2-(Cyclopropylmethylene)hydrazinyl)-4-(4-nitrophenyl)thiazole (**3j**)

2-Bromo-1-(4-nitrophenyl)ethanone was reacted with **2**. Yield: 0.56 g, 98%, (dichloromethan, *R*_f_ = 0.24); mp 168–171 °C. ^1^H NMR (DMSO-d_6_, 400 MHz); *δ* (ppm): 0.64–0.70 (m, 2H, CH_2_); 0.82–0.88 (m, 2H, CH_2_); 1.51–1.62 (m, 1H, CH); 6.93 (d, 1H, CH, *J* = 7.72 Hz); 7.61 (s, 1H, CH); 8.07 (d, 2H, 2CH, *J* = 9.0 Hz); 8.25 (d, 2H, 2CH, *J* = 9.0 Hz); 11.64 (bs, 1H, NH). ^13^C NMR (DMSO-d_6_, 100 MHz): *δ* (ppm) 6.49 (2C_cyclopropyl_); 14.02 (C_cyclopropyl_); 108.19 (C_thiazole_); 124.47 (2C_Ar_); 126.81 (2C_Ar_); 140.85 (C); 146.61 (C); 148.21 (C_thiazole_); 150.55 (C=N); 169.25 (C–NH). GC-EI-MS (*m*/*z*, %): 288 [(M^+^), 45], 221 (100), 174 (40). Anal. calcd. for C_13_H_12_N_4_O_2_S: C, 54.15; H, 4.20; N, 19.43. Found: C, 54.15; H, 4.21; N, 19.45.

### Microbiology

The examined compounds **3a**–**3j** were screened in vitro for antibacterial and antifungal activities using the broth microdilution method according to European Committee on Antimicrobial Susceptibility Testing (EUCAST) (EUCAST 2003) and Clinical and Laboratory Standards Institute guidelines (CLSI 2012) against reference strains of microorganisms from American Type Culture Collection (ATCC), including fungi belonging to yeasts (*Candida albicans* ATCC 2091, *Candida albicans* ATCC 10231, *Candida parapsilosis* ATCC 22019 and *Candida krusei* ATCC 14243).

In the study of antifungal activity of the compounds **3a**–**3j**, 15 clinical strains of different species of yeasts from *Candida* species, namely *C. albicans*, *C. dubliniensis C. famata, C. inconspicua, C. krusei, C. tropicalis, C. glabrata, C. lambica, C. kefyr, C. lusitaniae, C. parapsilosis, C. guilliermondii, C. lusitaniae, C. pulcherrima* and *C. sake* were also used. These fungi were isolated by the author (from Department of Pharmaceutical Microbiology of Medical University in Lublin, Poland) from different clinical materials, e.g., from upper respiratory tract of hospitalized patients including cancer patients (i.e., with non-small cell lung cancer or hematological malignancies). Some patients were after pre-operative or post-operative chemotherapy (treated with etoposide or *cis*-platin given in doses according to the standard procedures), patients with chronic hepatitis C (undergoing peginterferon and ribavirin therapy or without antiviral therapy), patients with diabetes, elderly people, aged of 65 years old or older, staying in close population, such as a care centre and people staying outside the home care. The Ethical Committee of the Medical University of Lublin approved the study protocol (No. KE-0254/75/2011). The isolates were identified by standard diagnostic methods—biochemical microtest, e.g., API 20C AUX, ID 32C, API Candida (bioMérieux) on the basis of assimilation of various substrates. All the used microbial cultures were first subcultured on nutrient agar or Sabouraud agar for bacteria and fungi, respectively. The RPMI 1640 with MOPS (for fungi) were inoculated with the suspensions of fungal species. Microbial suspensions were prepared in sterile saline (0.85% NaCl) with an optical density of 0.5 McFarland standard scale (Wiegand et al. 2008). Samples containing examined compounds were dissolved in dimethyl sulfoxide (DMSO). Furthermore, fungal suspensions were put onto Petri dishes with solid media containing 2 mg/ml of the tested compounds followed incubation under appropriate conditions. The inhibition of microbial growth was judged by comparison with a control culture prepared without any sample tested. Nystatin (Sigma) was used as a reference antifungal compound. Subsequently MIC minimal inhibitory concentration (MIC) of the compounds was examined by the microdilution broth method, using their two-fold dilutions in RPMI 1640 broth with MOPS prepared in 96-well polystyrene plates. Final concentrations of the compounds ranged from 1000 to 0.0038 µg/ml. Microbial suspensions were prepared in sterile saline with an optical density of 0.5 McFarland standard. Next fungal suspension was added per each well containing broth and various concentrations of the examined compounds. After incubation, the MIC was assessed spectrophotometric as the lowest concentration of the samples showing complete fungal growth inhibition. Appropriate DMSO, growth and sterile controls were carried out. The medium with no tested substances was used as a control (Wiegand et al. 2008). The minimal fungicidal concentration (MFC) are defined as the lowest concentration of the compounds that is required to kill a particular fungal species. MFC was determined by removing the culture using for MIC determinations from each well and spotting onto appropriate agar medium. The plates were incubated. The lowest compounds concentration with no visible growth observed was assessed as a fungicidal concentration. All the experiments were repeated three times and representative data are presented.

In this study, no bioactivity was defined as a MIC > 1000 µg/ml, mild bioactivity as a MIC in the range 501–1000 µg/ml, moderate bioactivity with MIC from 126 to 500 µg/ml, good bioactivity as a MIC in the range 26–125 µg/ml, strong bioactivity with MIC between 10 and 25 µg/ml and very strong bioactivity as a MIC < 10 µg/ml. The MFC/MIC ratios were calculated in order to determine fungicidal (MFC/MIC ≤ 4) or fungistatic (MFC/MIC > 4) effect of the tested compounds (O’Donnell et al. 2010).

### In vivo pharmacology

#### Animals

For in vivo tests that assessed anticonvulsant properties of the test compounds **3a**–**3j** adult male Albino Swiss (CD-1) mice weighing between 18 and 22 g were used. The animals were housed in groups of ten mice per cage at room temperature of 22 ± 2 °C, under light/dark (12:12) cycle. The animals had free access to food and tap water before the experiments. The ambient temperature of the experimental room and humidity (50 ± 10%) were kept consistent throughout all the tests. For behavioral experiments the animals were selected randomly. Each experimental group consisted of 4–8 animals/dose. The experiments were performed between 9 AM and 2 PM. Immediately after the in vivo assay the animals were euthanized by cervical dislocation. All procedures were approved by the Local Ethics Committee of the Jagiellonian University in Krakow (34/2018, 1.02.2018)) and the treatment of animals was in full accordance with ethical standards laid down in respective Polish and EU regulations (Directive No. 86/609/EEC).

#### Chemicals used in pharmacological tests

For in vivo tests the compounds **3a**–**3j** were prepared in 1% Tween 80 solution (POCH, Poland) and they were administered by the intraperitoneal route. Control mice received 1% Tween 80. Pentylenetetrazole (PTZ), pilocarpine hydrochloride and scopolamine butylbromide were provided by Sigma Aldrich (Poland). For the tests they were prepared in 0.9% saline (Polfa Kutno, Poland). PTZ and pilocarpine were administered 60 min after the test compound or vehicle. To assess anticonvulsant properties of the test compounds anticonvulsant screening was conducted. Four assays were used: PTZ, maximal electroshock seizure (MES), 6-Hz and pilocarpine tests. In these assays the dose of 100 mg/kg was chosen as a starting dose. If it turned out to be effective, a lower dose (30 mg/kg) was also tested.

#### PTZ seizure test

The test was performed according to a method previously described (Sałat et al. 2005). Clonic convulsions were induced by the subcutaneous (sc) administration of PTZ at a dose of 100 mg/kg. After PTZ injection, each mouse was immediately placed in a transparent Plexiglas cage (30 × 20 × 15 cm) and was observed during the next 30 min for the occurrence of clonic seizures. Clonic seizures were defined as clonus of the whole body lasting more than 3 s, with an accompanying loss of righting reflex. Latency time to first clonus and the number of seizure episodes were noted and compared between vehicle-treated and drug-treated groups.

#### Maximal electroshock seizure test

MES test was performed according to a method previously described (Sałat et al. 2005). In this test vehicle-treated mice and drug-treated mice received a stimulus of 25 mA delivered by an electroshock generator (Hugo Sachs rodent shocker, Germany) to induce maximal seizures (tonic extension) of hind limbs. Electroconvulsions were produced with the use of auricular electrodes and the stimulus duration was 0.2 s. Tonic extension of the hind limbs was regarded as the endpoint for this procedure.

#### 6-Hz test

This test was performed according to (Barton et al. 2001). It is an alternative electroshock paradigm that involves low-frequency (6 Hz), long-duration (3 s) electrical stimulation. Corneal stimulation (0.2 ms-duration monopolar rectangular pulses at 6 Hz for 3 s) was delivered by a constant-current device. During electrical stimulation mice were manually restrained and released into the observation cage immediately after current application. At the time of drug administration a drop of 0.5% tetracaine (Altacaine sterile solution, Altaire Pharmaceuticals Inc., USA) was applied into the eyes of all animals. Prior to the placement of corneal electrodes, a drop of 0.9% saline was applied on the eyes. In this model seizures manifest in ‘stunned’ posture associated with rearing, forelimb automatic movements and clonus, twitching of the vibrissae and Straub-tail. At the end of the seizure episode the animals resume their normal exploratory behavior. In this test protection against a seizure episode is considered as the end point and animals are considered to be protected if they resume their normal exploratory behavior within 10 s after electrical stimulation.

#### Pilocarpine-induced seizures

In this test the mice were pretreated with the investigated compound or vehicle and 60 min later they received pilocarpine (400 mg/kg, ip). To avoid cholinergic side-effects: peripheral toxicity and diarrhea, masticatory and stereotyped movements, animals treated with pilocarpine also received scopolamine butylbromide (1 mg/kg, ip) which was injected 45 min before pilocarpine. After the administration of the convulsant, the mice were observed during the next 60 min for behavioral changes. Latency time to the onset of *status epilepticus* was considered as the endpoint in this test (Wilhelm et al. 2010).

#### Rotarod test

The test was performed according to the method recently described (Sałat et al. 2005). The mice were trained daily for 3 consecutive days on the rotarod apparatus (Rotarod apparatus, May Commat RR0711, Turkey; rod diameter: 2 cm) rotating at a constant speed of 18 rotations per minute (rpm). During each training session, the animals were placed on a rotating rod for 3 min with an unlimited number of trials. The proper experimentation was conducted 24 h after the final training trial. Briefly, 60 min before the rotarod test the mice were pretreated with the test compound (100 mg/kg) and then, they were tested on the rotarod apparatus revolving at 6, 18, and 24 rpm. Motor impairments, defined as the inability to remain on the rotating rod for 1 min were measured and mean time spent on the rod was counted in each experimental group.

#### Data analysis

Data analysis of the results obtained in behavioral tests was provided by GraphPad Prism Software (ver. 5, CA, USA). The results were statistically evaluated using one-way analysis of variance (ANOVA), followed by Dunnett’s post-hoc comparison. *P* < 0.05 was considered significant.

### Antiparasitic activity

#### Cell and parasite culture

L929 cell line (ATCC® CCL-1™) was cultured in IMDM (IMDM/10%FBS/P/S) (Iscove’s Modified Dulbecco’s Medium—Biowest) culture medium with the addition of 10% FBS (Fetal Bovine Serum—Biowest), 100 μg/ml streptomycin (Sigma), 100 U/ml penicillin (Sigma). Vero cell line (ATCC® CCL-81^™^) were maintained in EMEM (EMEM/10%FBS/P/S) (Eagle’s Minimum Essential Medium ATCC® 30-2003™) culture medium supplemented with 10% FBS, 100 μg/ml streptomycin, 100 U/ml penicillin. Both cells line were trypsinized twice a week, then seeded in T25 cell culture flask [Falcon], density 1 × 10^6^/flask and incubated for 24–48 h at 37 °C and 5 or 10% CO_2_ to achieve a confluent monolayer. Female BALB/cW mice, aged 8–12 weeks were bred as homozygotes, under conventional conditions in the animal facility of the Faculty of Biology and Environmental Protection, University of Lodz. Animal experiments were conducted according to guidelines provided by the Polish Local Ethics Commission for Experiments on Animals No. 9 in Lodz (agreement 67/ŁB80/2017). Tachyzoites of *Toxoplasma gondii* (*Tg*) virulent RH strain (ATCC® 50174^™^) were propagate by inteperitional inoculation of mice, dose per mouse 5 × 10^6^ *Tg* tachyzoite in 200 μl of PBS (PBS/5%FBS/P/S) (Dulbecco’s Phosphate Buffered Saline, w/o magnesium and calcium—Biowest) supplemented with 5% FBS, 100 μg/ml streptomycin, 100 U/ml penicillin. The mice were sacrificed after 3–4 days by the cervical dislocation, and then parasites were harvested from peritoneal cavity by lavage with 10 ml of PBS/5%FBS/P/S and counted using haemocytometer. For further testing density of *Tg* tachyzoites was adjusted to 1 × 10^6^/ml in IMDM (IMDM/5%FBS/P/S) medium supplemented with 5% FBS, 100 μg/ml streptomycin, 100 U/ml penicillin (Dzitko et al. 2014).

#### Compounds and drug preparation for MTT and [^3^H]-uracil incorporation assay

Compounds (**3a**–**3j**) were dissolved in dimethyl sulfoxide (DMSO) (Sigma) 5 mg/ml. Final concentration of DMSO was not higher than 0.625% in IMDM/5%FBS/P/S or RPMI 1640 without phenol red (Biowest) (RPMI(w/oPhR)/ 10%FBS/P/S) culture medium supplemented with 10% FBS, 100 μg/ml streptomycin, 100 U/ml penicillin. Sulfadiazine (S8626, Sigma) was dissolved in 1 M sodium hydroxide (NaOH) (Sigma) 100 mg/ml. Final concertation of NaOH was not higher than 2.5% in IMDM/5%FBS/P/S.

#### [^3^H]-Uracil incorporation assay

Vero cells were seeded in a 96-well tissue culture plate (Falcon) 100 μl/well from density 1 × 10^5^/ml and incubated for 24 h at 37 °C and 5% CO_2_ to achieve a confluent monolayer. Afterwards, the culture medium was replaced with 100 μl per well 1 × 10^6^/ml *Tg* in IMDM/5%FBS/P/S and incubated for 1 h at 37 °C and 10% CO_2_. After incubation 100 μl/well solutions of the compounds and drug were added. The dilutions of tested compounds (final concentrations range 0.00–31.25 μg/ml) and sulfadiazine (final concentrations range 0.00–2500.00 μg/ml) were prepared in IMDM/5%FBS/P/S. Then, plates were incubated for a further 48 h under the previously mentioned conditions. After that, 25 μl/well of [5,6-^3^H]-uracil (42.3 Ci/mmol) (PerkinElmer) was added, and incubated for 20 h at 37 °C and 10% CO_2_. Plates were frizzed at −20 °C for a next 24 h, and then thawed in room temperature, cells and supernatants were harvested on glass fiber filters (Wallac Oy) and counted in scintillation counter (Wallac Oy, Turku, Finland). The results were presented as a percentage (%) of proliferated parasite and the half maximal inhibitory concentration (IC_50_) anti-*Tg* were established. Each experiment was performed in triplicate. Statistical analyses and graphs were performed using GraphPad Prism version 7.00 for Windows (GraphPad Software, San Diego, CA, USA) (Dzitko et al. 2014).

#### Cytotoxicity evaluation

Cytotoxicity was evaluated using the MTT assay, which is based on the ability of live cells to convert the water-soluble tetrazolium salt (MTT) ((3-(4,5-Dimethylthiazol-2-yl)-2,5-diphenyltetrazolium bromide)—Sigma) to the water-insoluble formazan crystals by the mitochondrial dehydrogenase enzyme. The colour intensity of the solution after dissolution of the crystals is measured spectrophotometrically and corresponds to cell viability. The MTT assay is the most popular test used for the evaluation of cytotoxic activity according to the ISO standard 10993-5:2009(E). The assay was performed using two cell lines, L929 (ATCC® CCL-1™) and Vero (ATCC® CCL-81™). Cells were lead to a density of 1 × 10^5^/ml, L929 in IMDM/10%FBS/P/S but Vero in EMEM/10%FBS/P/S, and their suspension was seeded in a 96-well tissue culture plate (Falcon) 100 μl/well and incubated for 24 h at 37 °C and 5 or 10% CO_2_ to achieve a confluent monolayer. Afterwards, the culture medium was replaced with solutions of the compounds in RPMI(w/oPhR)/10%FBS/P/S medium at concentrations of 0–31.25 μg/ml and the cells were incubated for a further 24 h at 37 °C and 5% CO_2_. Further 5 mg/ml MTT solution was prepared and added to the culture in a volume of 10 μl/well. The plates prepared in this way were incubated under the previously mentioned conditions for 4 h. After incubation, the plates were centrifuged for 15 min at 2500 rpm, then the supernatants were removed. The resulting formazan crystals were dissolved by adding 150 μl DMSO to each well, the plates were gently mixed, then 25 μl 0.1 M glycine buffer (pH = 10.5) (Sigma) was added. Optical density was measured using the ELISA plate reader (Multiskan EX, Labsystems, Vienna, VA, USA) at 570 nm. The results were presented as a percentage (%) of viable cells. Each experiment was performed in triplicate.

### Automated docking setup

Flexible docking was performed by means of the FlexX (Kramer et al. 1999) program as implemented in LeadIT software package (LeadIT 2012). Models of the secreted aspartic proteinase (SAP), and *N*-myristoyltransferase (NMT), and calcium channel receptor binding sites based on the structure deposited in the Protein Data Bank under the 1EAG, 1IYL, and 5IWP were employed. The active sites were defined to include all atoms within 6.5 Å radius of the native ligands. The first 100 top ranked docking poses were saved for each docking run. For all compounds their protonated forms were considered, as recommended by FlexX program.

## Results and discussion

### Chemistry

**Scheme 1:**
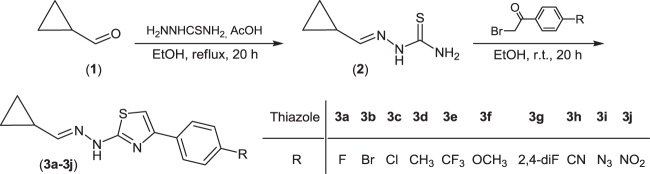
Synthesis of the target compounds **3a**–**3j**

2-(Cyclopropylmethylidene)hydrazinecarboxamide (**2**) was readily synthesized in high yield by heating of ethanolic solution of cyclopropanecarboxaldehyde (**1**) and thiosemicarbazide containing catalytic amount of glacial acetic acid. In the next step Hantzsch cyclization reaction of hydrazinecarbothioamide **2** with appropriate *para*-substituted bromoacetophenones in ethanolic solution and under room temperature produced (2-(cyclopropylmethylidene)-hydrazinyl)thiazoles **3a**–**3j** with high yield (52–99%) and with high chemical purity. The reaction pathway has been summarized in Scheme 1. All obtained products were purified on silica gel column chromatography, and fully characterized spectroscopically using ^1^H and ^13^C NMR, GC-EI-MS, and elemental analyses.

In the ^1^H NMR spectrum of compound **2**, three characteristic signals derived from the NH_2_ and NH groups at 7.44, 7.88, and 10.98 ppm can be observed. These three signals are the result of the exchange of a hydrogen atom between the NH_2_ group and the sulfur atom, and is a characteristic feature for hydrazinecarbothioamides. Also ^1^H NMR spectra of (2-(cyclopropylmethylidene)hydrazinyl)thiazole (**3a**–**3j**) showed characteristic singlet at *δ* (7.06–7.61) ppm due to the presence of the H-5 atom in the thiazole ring and broadened hydrazine NH singlet at *δ* (11.47–11.64) ppm. The ^13^C NMR of carbon atoms present in C=N group resonates around 150 ppm which proves that the conversion of substrates to the expected products was successful. The [M^+^] peaks were observed in the mass spectra of all compounds, confirming the assigned structures. Purity of the products was confirmed by the elemental analyses, whose results were in good agreement with the calculated values. All reactions were repeated at least two times and are fully reproducible.

### Biological evaluation

#### Antifungal activity

On the basis of MIC obtained by the broth microdilution method, it was shown that compounds **3a**–**d** and **3f**–**j** possess strong or very strong activity against *Candida* spp. ATCC with MIC = 0.015–7.81 µg/ml (Table 1) and MFC = 0.03–31.25 µg/ml (Table 2). In addition, most substances exhibited fungicidal activity (MFC/MIC = 1–4), and some of them showed fungistatic effect (MFC/MIC = 8–16) (Table 2). The activity of these compounds is similar and even higher than the activity of nystatin used as positive control.

**Table 1:** The activity data expressed as MIC (µg/ml) against the reference and clinical strains of fungi

Species	MIC (µg/ml) of the tested compounds									
	3a	3b	3c	3d	3f	3g	3h	3i	3j	Nystatin
*C. albicans* ATCC 2091	1.95	0.12	0.12	0.12	0.24	1.95	1.95	0.98	3.91	0.24
*C. albicans* ATCC 10231	1.95	0.06	0.12	0.06	0.015	0.48	0.48	0.24	0.48	0.48
*C. parapsilosis* ATCC 22019	1.95	0.48	0.48	0.24	0.48	3.91	3.91	0.98	3.91	0.24
*C. krusei* ATCC 14243	3.91	0.24	0.12	0.06	0.48	7.81	0.98	0.48	0.98	0.24
*C. krusei*	0.98	0.24	0.12	0.24	0.015	0.03	0.48	0.98	0.06	0.24
*C. tropicalis*	0.98	0.06	0.12	0.06	0.06	0.06	0.03	0.24	0.015	0.015
*C. inconspicua*	1.95	0.24	0.98	0.12	0.03	0.48	0.24	0.48	0.98	0.12
*C. famata*	7.81	0.12	0.12	0.48	0.48	7.81	1.95	1.95	1.95	0.06
*C. guilliermondii*	1.95	0.12	0.24	0.48	0.48	7.81	0.48	0.98	0.24	0.06
*C. lusitaniae*	0.98	0.98	0.12	0.12	0.12	0.48	0.24	0.48	0.48	0.12
*C. sake*	3.91	0.06	0.12	0.12	0.06	0.48	0.98	0.48	0.24	0.06
*C. dubliniensis*	1.95	0.06	0.03	0.06	0.015	0.24	0.48	0.015	0.24	0.12
*C. parapsilosis*	3.91	0.12	0.03	0.06	0.12	0.98	1.95	0.24	0.48	0.12
*C. albicans*	0.98	0.12	0.06	0.12	0.24	0.48	0.98	0.48	0.24	0.24
*C. kefyr*	–	15.62	31.25	1.95	1.95	31.25	7.81	7.81	125	0.12
*C. pulcherrima*	–	–	125	15.62	31.25	250	62.5	62.5	7.81	0.12
*C. glabrata*	–	62.5	500	15.62	31.25	15.62	125	31.25	125	0.48
*C. lambica*	–	–	125	15.62	31.25	125	125	–	250	0.48

**Table 2:** The activity data expressed as MFC (µg/ml) and (MFC/MIC) against the reference and clinical strains of fungi

Species	MFC (µg/ml) and (MFC/MIC) of the tested compounds									
	3a	3b	3c	3d	3f	3g	3h	3i	3j	Nystatin
*C. albicans* ATCC 2091	3.91 (2)	0.98 (8)	0.48 (4)	1.95 (16)	1.95 (8)	1.95 (1)	3.91 (2)	3.91 (4)	7.81 (2)	0.24 (1)
*C. albicans* ATCC 10231	15.62 (8)	0.24 (4)	0.24 (2)	0.24 (4)	0.03 (2)	0.98 (2)	1.95 (4)	1.95 (8)	1.95 (4)	0.48 (1)
*C. parapsilosis* ATCC 22019	3.91 (2)	1.95 (4)	1.95 (4)	0.98 (4)	3.91 (8)	3.91 (1)	3.91 (1)	7.81 (8)	31.25 (8)	0.48 (2)
*C. krusei* ATCC 14243	15.62 (4)	0.98 (4)	0.98 (8]	0.48 (8)	0.98 (2)	31.25 (4)	1.95 (2)	1.95 (4)	3.91 (4)	0.24 (1)
*C. krusei*	1.95 (2)	0.48 (2)	0.48 (4)	0.98 (4)	0.015 (1)	0.48 (16)	7.81 (16)	7.81 (8)	0.12 (2)	0.98 (4)
*C. tropicalis*	1.95 (2)	0.12 (2)	0.12 (1)	0.12 (2)	0.98 (16)	0.98 (16)	0.98 (32)	1.95 (8)	0.015 (1)	0.06 (4)
*C. inconspicua*	3.91 (2)	3.91 (16)	3.91 (4)	0.98 (8)	0.98 (32)	1.98 (4)	0.48 (2)	0.98 (2)	1.95 (2)	0.48 (4)
*C. famata*	62.5 (8)	0.98 (8)	0.98 (8)	3.91 (8)	7.81 (16)	62.5 (8)	3.91 (2)	7.81 (4)	31.25 (16)	0.12 (2)
*C. guilliermondii*	31.25 (16)	0.48 (4)	0.98 (4)	3.91 (8)	7.81 (16)	31.25 (4)	3.91 (8)	15.62 (16)	0.98 (4)	0.12 (2)
*C. lusitaniae*	3.91 (4)	1.95 (2)	0.12 (1)	0.48 (4)	0.48 (4)	1.95 (4)	0.98 (4)	1.95 (4)	0.98 (2)	0.24 (2)
*C. sake*	15.62 (4)	0.24 (4)	0.98 (8)	0.48 (4)	7.81 (128)	3.91 (8)	125 (128)	7.81 (16)	0.98 (4)	0.12 (2)
*C. dubliniensis*	3.91 (2)	0.06 (1)	0.06 (2)	0.12 (2)	0.06 (4)	0.48 (2)	0.98 (2)	0.12 (2)	0.48 (2)	0.12 (1)
*C. parapsilosis*	7.81 (2)	0.12 (1)	0.12 (4)	0.24 (4)	0.98 (8)	1.95 (2)	31.25 (16)	0.98 (4)	1.95 (4)	0.48 (4)
*C. albicans*	3.91 (4)	1.95 (16)	0.98 (16)	0.24 (2)	0.98 (4)	1.95 (4)	15.62 (16)	15.62 (32)	0.48 (2)	0.24 (1)
*C. kefyr*	–	>1000 (nd)	>1000 (nd)	7.81 (4)	7.81 (4)	31.25 (1)	125 (16)	>1000 (nd)	>1000 (nd)	0.24 (2)
*C. pulcherrima*	–	–	>1000 (nd)	>1000 (nd)	>1000 (nd)	>1000 (nd)	>1000 (nd)	>1000 (nd)	>1000 (nd)	0.24 (2)
*C. glabrata*	–	>1000 (nd)	>1000 (nd)	>1000 (nd)	>1000 (nd)	>1000 (nd)	>1000 (nd)	>1000 (nd)	>1000 (nd)	0.48 (1)
*C. lambica*	–	–	>1000 (nd)	>1000 (nd)	>1000 (nd)	>1000 (nd)	>1000 (nd)	–	>1000 (nd)	0.98 (2)

The tested compounds **3a**–**d** and **3f**–**j** possess very strong activity towards most of strains of *Candida* spp. isolated from clinical materials. (namely *C. albicans*, *C. dubliniensis C. famata, C. inconspicua, C. krusei, C. tropicalis, C. lusitaniae, C. parapsilosis, C. guilliermondii, C. lusitaniae*, and *C. sake*) with the same value of MIC = 0.015–7.81 µg/ml and MFC = 0.015–125 µg/ml (Table 2). The antifungal effect was both fungicidal (MFC/MIC = 1–4) and fungistatic (MFC/MIC = 8–128) (Table 3). The activity of these substances was also similar to nystatin. However, these compounds indicated weaker or none activity against other clinical isolates of Candida, such as *C. kefyr*, *C. pulcherrima*, *C. glabrata*, and *C. lambica*. The strong to medium activity showed only compounds **3d**, **3f**, **3h**, and **3i** towards *C. kefyr* and **3j** towards *C. pulcherrima* with MIC = 1.95–125 µg/ml.

**Table 3:** Anticonvulsant activity of compounds **3a**–**3j** in MES test

Compound	Dose (mg/kg)	*X*/*Y*^a^
Vehicle	–	0/6
**3a**	100	3/8
**3b**	100	3/8
**3c**	30	0/8
	100	6/8
**3d**	100	3/8
**3e**	30	0/8
	100	5/8
**3f**	100	4/8
**3g**	100	3/8
**3h**	100	4/8
**3i**	100	3/8
**3j**	100	4/8

^a^Results are shown as number of mice protected (*X*) per number of mice tested (*Y*)

In addition, compound **3e** was found to be inactive against all tested strains of Candida, as well as, all compounds were inactive towards *C. glabrata* ATTC 90030, which is naturally insensitive to azole-derived antifungal agents (Tumbarello et al. 2008).

The structure-activity relationship (SAR) analysis showed that in the case of **3a**–**3d** and **3f**–**3j** derivatives, the type of substituent does not affect the activity. However, it can be seen that compound **3e** containing the trifluoromethyl substituent did not show any activity, and the only explanation of this fact is that this group is sterically hindered.

#### Anticonvulsant activity

**Fig. 1:**
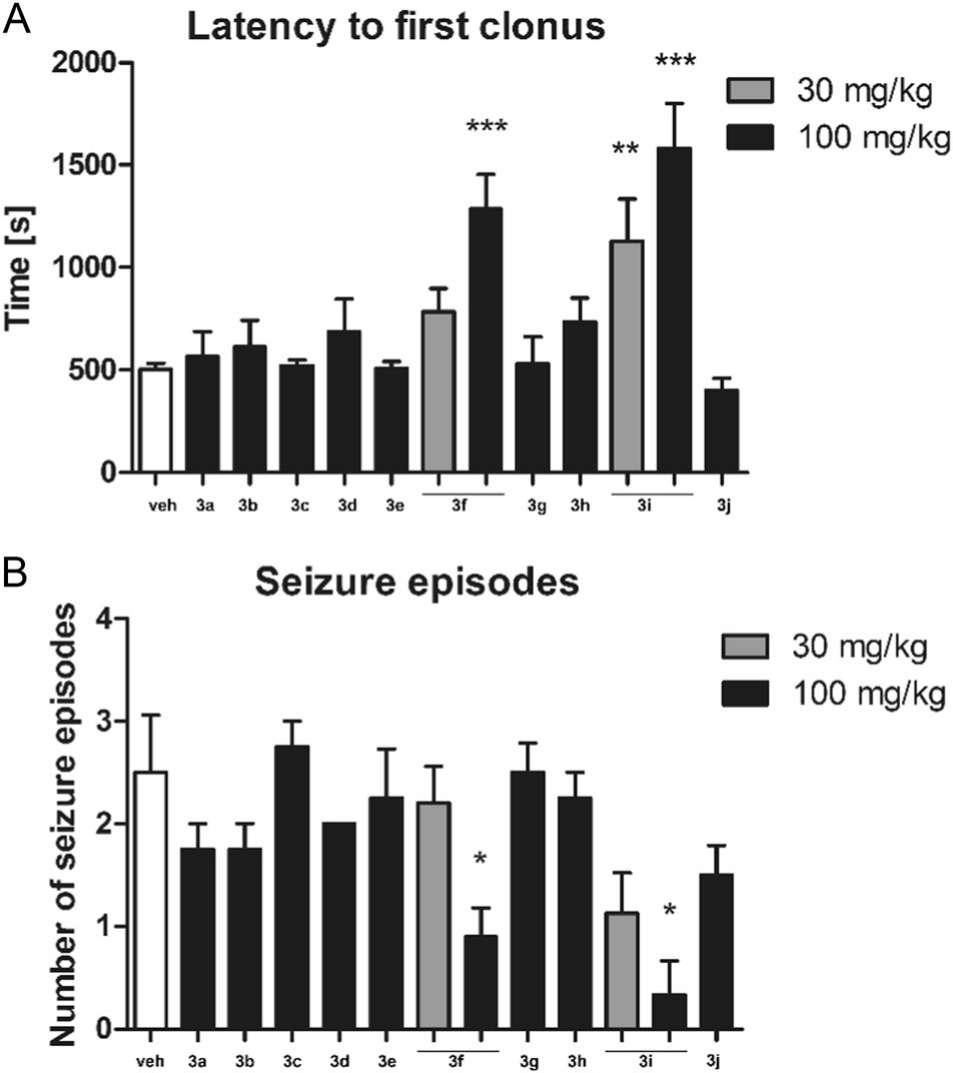
Influence of the test compounds **3a**–**3j** on latency time to first clonus **a** and the number of seizure episodes **b** in PTZ-induced seizure model. Statistical analysis: one way analysis of variance (ANOVA), followed by Dunnett’s post-hoc test. Significance vs. vehicle-treated mice: *p < 0.05, **p < 0.01, ***p < 0.001

In the PTZ model of chemically-induced seizures an overall effect of treatment on latency time to first clonus (F[12,58] = 4.967, *p* < 0.0001) and number of seizure episodes (F[12,56] = 3.137, *p* < 0.01) was observed. Two compounds: **3f** and **3i** showed anticonvulsant properties and **3i** was more efficacious in this respect. These two agents significantly prolonged latency time to first clonus (Fig. 1a) and reduced the number of seizure episodes (Fig. 1b). Other compounds tested were not effective in this assay.

The compounds **3c** and **3e** demonstrated the highest anticonvulsant activity in MES test. At the dose 100 mg/kg they reduced seizures in 75% and 63% of animals, respectively, but the dose 30 mg/kg of each compound was not active (Table 3). Other compounds tested at 100 mg/kg showed lower efficacy in the MES test.

In the 6-Hz test the anticonvulsant activity of the test compounds was marginal (Table 4). Only the compound **3i** at the dose of 100 mg/kg protected 75% of animals from seizures in this test.

**Table 4:** Anticonvulsant activity of compounds **3a**–**3j** measured in 6-Hz test

Compound	Dose (mg/kg)	*X*/*Y*^a^
Vehicle	–	1/4
**3a**	100	1/4
**3b**	100	0/4
**3c**	100	1/4
**3d**	100	2/4
**3e**	100	1/4
**3f**	100	2/4
**3g**	100	1/4
**3h**	100	2/4
**3i**	30	1/4
	100	3/4
**3j**	100	0/4

^a^Results are shown as number of mice protected (*X*) per number of mice tested (*Y*)

**Fig. 2:**
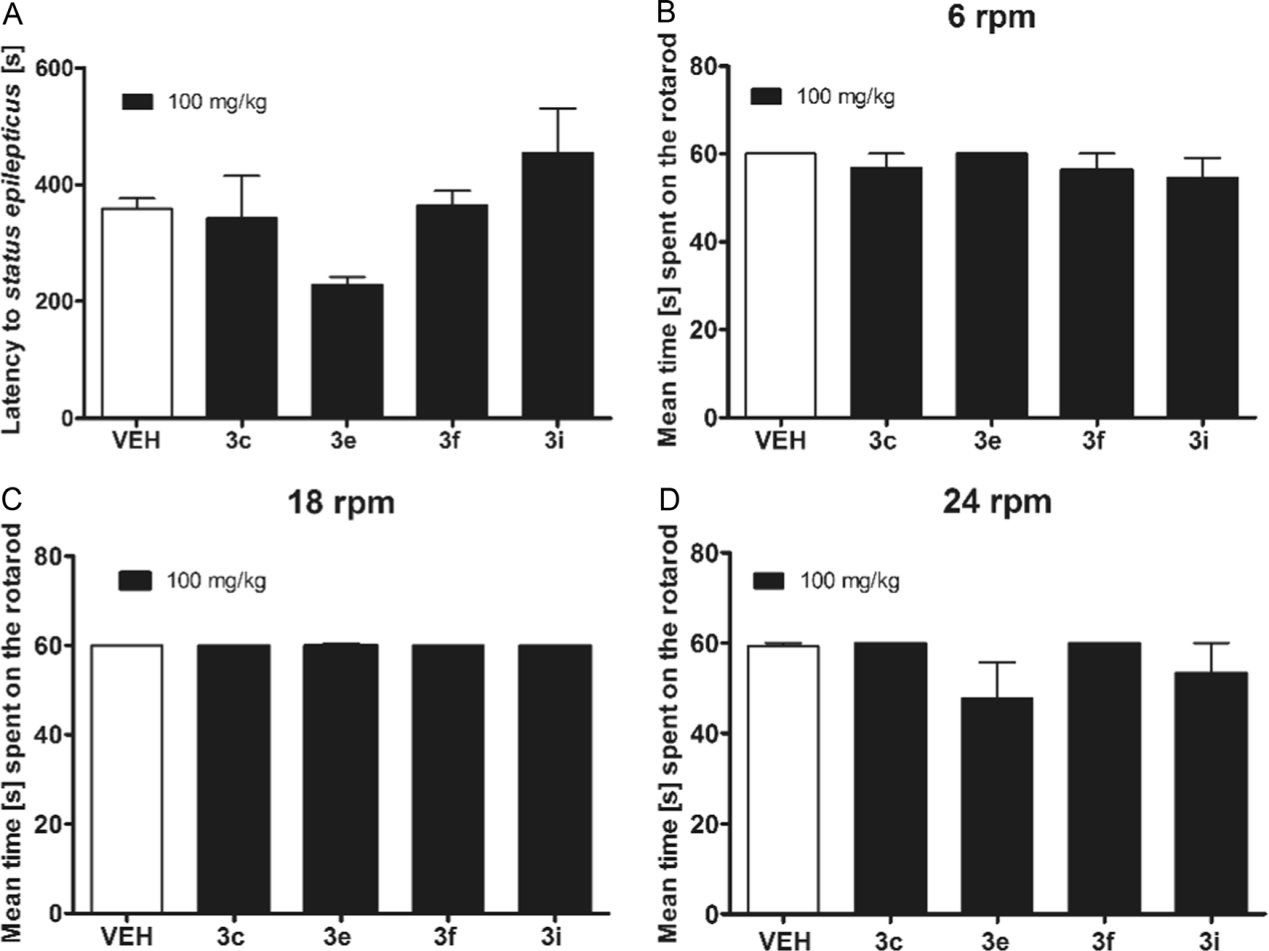
Influence of the compounds **3c**, **3e**, **3f**, and **3i** at the dose of 100 mg/kg on: **a** latency time to pilocarpine-induced *status epilepticus*, or **b**–**d** motor coordination measured in the rotarod test. Results are shown as latency time to *status epilepticus* (±SEM), or mean time spent on the rotarod (±SEM) rotating at 6, 18, or 24 rpm. Statistical analysis: one-way analysis of variance (ANOVA), followed by Dunnett’s post-hoc comparison: *p* > 0.05

Four compounds which showed the highest anticonvulsant activity in PTZ, MES or 6-Hz tests: **3c**, **3e**, **3f**, and **3i** were also assessed for their potential anticonvulsant activity in pilocarpine-induced seizure model. One-way ANOVA revealed an overall effect of treatment (F[4,37] = 2.747, *p* < 0.05) but the post-hoc analysis did not show statistically significant anticonvulsant effects of the compounds tested (Fig. 2a).

Four compounds: **3c**, **3e**, **3f**, and **3i** (100 mg/kg) which showed protection in PTZ, MES, or 6-Hz tests were additionally tested in the rotarod test to assess their impact on animals’ motor coordination. None of these compounds impaired motor skills of experimental animals (Fig. 2b-d) and all animals were able to perform the test at 6 rpm (F[4,35] = 0.6383, *p* > 0.05), 18 rpm (F[4,35] = 1.000, *p* > 0.05) and 24 rpm (F[4,35] = 1.348, *p* > 0.05).

The main goal of the in vivo part of this study was to assess anticonvulsant properties of (2-(cyclopropylmethylidene)-hydrazinyl)thiazole **3a**–**3j** in mouse models of seizures. For this purpose we used four distinct screening assays, i.e., MES, 6-Hz and PTZ and pilocarpine tests. These tests are regarded “gold standards” in the search for novel antiepileptic drugs and they allow to predict the efficacy of anticonvulsant active agents against different types of seizures in humans (Loescher 2011). The MES test is useful for selection of drugs for generalized tonic-clonic seizures, 6-Hz psychomotor seizure model resembles psychomotor seizures occurring in human limbic epilepsy (Loescher 2011; Loescher and Schmidt 2011) and the PTZ model of clonic seizures generally refers to non-convulsive (*absence* or myoclonic) seizures in humans (Loescher 2011). Pilocarpine model enables to select compounds able to cease *status epilepticus* and drug-resistant epilepsy (Blanco et al. 2009).

The results obtained in the present study demonstrated a distinct anticonvulsant activity of the test compounds. In MES test two compounds—**3c** and **3e** revealed a higher anticonvulsant activity than other agents tested. Noteworthy, they were not effective in the PTZ model. This might indicate for a potential usefulness of **3c** and **3e** in tonic-clonic seizures (*grand mal* epilepsy) in humans but not in *absence* seizures. This finding also suggests that their mechanism of action is likely to be related to the blockade of voltage-gated sodium channels but not to the inhibition of T-type calcium channels (Loescher 2011; Loescher and Schmidt 2011; Brodie et al. 2011).

In contrast to this, the compounds **3f** and **3i** significantly delayed the onset of PTZ-induced seizures and they reduced the number of seizure episodes in PTZ-treated mice. It has been suggested that compounds showing activity in PTZ test might be useful in human *absence* seizures but not in *grand mal* epilepsy and their mechanism of action might be similar to that of ethosuximide—an antagonist of T-type voltage-gated calcium channels (Loescher 2011; Loescher and Schmidt 2011). The compound **3i** was also effective in the mouse model of psychomotor seizures, i.e., the 6-Hz test. To some degree, this anticonvulsant activity of **3i** resembles that of levetiracetam. It should be however emphasized that **3i** was effective in both PTZ and 6-Hz test, while levetiracetam is active in only in 6-Hz test but not in PTZ test (Loescher 2011; Loescher and Schmidt 2011). Pilocarpine evokes spontaneous recurrent seizures that mimic *status epilepticus* in humans and is regarded as a very useful tool to study drug-resistant epilepsy (Blanco et al. 2009). The four compounds tested did not show protective properties against pilocarpine-induced seizures.

Motor coordination deficits are one of the most frequent adverse effects of available antiepileptic drugs and this effect might be a serious limitation of antiepileptic pharmacotherapy. Using the rotarod test we demonstrated that the test compounds at the anticonvulsant active dose do not induce any motor deficits in experimental animals.

To conclude, in the in vivo part of the present study we selected four compounds that showed anticonvulsant activity in screening tests in mice without impairing their motor skills. The compounds **3f** and **3i** demonstrated the highest anticonvulsant activity in PTZ-induced seizures and **3i** was also effective in 6-Hz test. In MES test the compounds **3c** and **3e** were active. Taken together, these compounds can be regarded as interesting novel lead structure in the search for novel anticonvulsant agents.

#### anti*-Toxoplasma gondii* activity

Due to the fact that available literature strongly suggests that a relationship between toxoplasmosis infection and epilepsy exists (Ngoungou et al. 2015; Palmer 2007), the prepared thiazoles **3a**–**3j** have been used to study intensity of *Toxoplasma gondii* virulent RH strain intracellular proliferation (%) in the VERO host cells. For this purpose, *Toxoplasma gondii* (tachyzoites) of RH strain were incubated with different concentrations of the thiazoles **3a**–**3j** ranging from 0.9 to 31.25 μg/ml. The parasite growth inhibition was monitored by measuring the specific incorporation of [^3^H]uracil in the parasite’s nucleic acids. The percentages of the parasite proliferation in VERO host cells by the compounds **3a**–**3j** and the control drug-sulfadiazine, as well as IC_50_ values are summarized in Table 5. According to these results, compounds **3a, 3h** and **3j** showed significant anti-*Toxoplasma gondii* activity, with IC_50_ values 31–52 times lower than those observed for sulfadiazine (IC_50_ = 935.8 μg/ml, see Supporting Information for more details). The highest activity showed compounds containing fluorine, cyano and nitro groups.

**Table 5:** Proliferation (%) of *Toxoplasma gondii* RH strain in the concentration range 0.98–31.25 μg/ml of tested compounds ± SD, and the half maximal inhibitory concentration (IC_50_) *anti-Tg*

Compounds concentration (µg/ml)	Compound									
	3a	3b	3c	3d	3e	3f	3g	3h	3i	3j
31.25	28.19 ± 5.7	nt	nt	nt	nt	68.03 ± 5.37	nt	43.81 ± 0.06	nt	35.09 ± 424
25.00	42.24 ± 0.91	nt	nt	nt	nt	57.79 ± 6.09	nt	53.86 ± 6.64	nt	40.27 ± 1.53
20.00	45.62 ± 5.64	nt	nt	nt	nt	58.53 ± 3.72	60.88 ± 3.81	56.59 ± 1.00	nt	58.76 ± 1.67
15.63	49.20 ± 3.59	nt	nt	84.41 ± 0.08	nt	63.04 ± 4.88	58.12 ± 2.94	64.98 ± 3.28	98.83 ± 4.29	59.97 ± 7.86
7.81	84.89 ± 0.78	111.25 ± 10.06	nt	90.44 ± 3.29	nt	86.20 ± 6.35	78.57 ± 4.60	75.46 ± 4.89	92.19 ± 3.71	96.70 ± 3.29
3.91	96.16 ± 0.61	106.75 ± 10.19	113.85 ± 6.65	93.38 ± 5.18	110.22 ± 4.37	79.17 ± 6.07	81.38 ± 1.60	79.64 ± 1.15	104.64 ± 2.24	97.14 ± 4.63
1.95	97.94 ± 7.49	99.32 ± 6.08	108.44 ± 4.34	100.26 ± 4.54	100.12 ± 2.25	84.19 ± 5.47	91.81 ± 1.12	83.26 ± 3.90	93.32 ± 4.67	94.44 ± 0.34
0.98	95.37 ± 2.41	85.81 ± 10.59	106.78 ± 0.02	88.66 ± 2.63	94.36 ± 6.92	89.61 ± 0.77	104.71 ± 9.98	86.14 ± 0.00	95.29 ± 10.58	90.98 ± 2.47
IC_50_ (µg/ml)	18.08	–	–	–	–	–	–	30.14	–	21.76

*SD* standard deviation, *nt* not tested because the stock compounds in DMSO after addition to culture medium crystallized, therefore the IC_50_ could not be determined

#### Cytotoxicity against mouse L929 fibroblast and African green monkey kidney VERO cells

The next stage of our research was to determine the toxicity of the newly synthesized compounds. This is a very important test because it determines whether the investigated compounds will be suitable for further clinical investigation. To demonstrate that these compounds are safe for host cells, we decided to investigate the cytotoxic effects of (2-(cyclopropylmethylidene)hydrazinyl)thiazole (**3a**–**3j**) on mouse L929 fibroblast, as well as the VERO cells using an MTT assay. The results of the cytotoxicity evaluation, *anti-Candida spp*. and anti-*Toxoplasma gondii* activity studies showed that *Candida spp*. and *Toxoplasma gondii* growth was inhibited at non-cytotoxic concentrations for the host cells (Table 6).

**Table 6:** The highest possible non-cytotoxic concentration of the compounds [µg/ml] tested on L929 and VERO cell lines

Thiazole	3a	3b	3c	3d	3e	3f	3g	3h	3i	3j
Conc.^a^	31.25	7.81	7.81	31.25	3.91	31.25	15.63	31.25	15.63	31.25

^a^Higher concentrations of the tested compounds could not be reached due to their limited solubility

### Molecular modelling studies

**Fig. 3:**
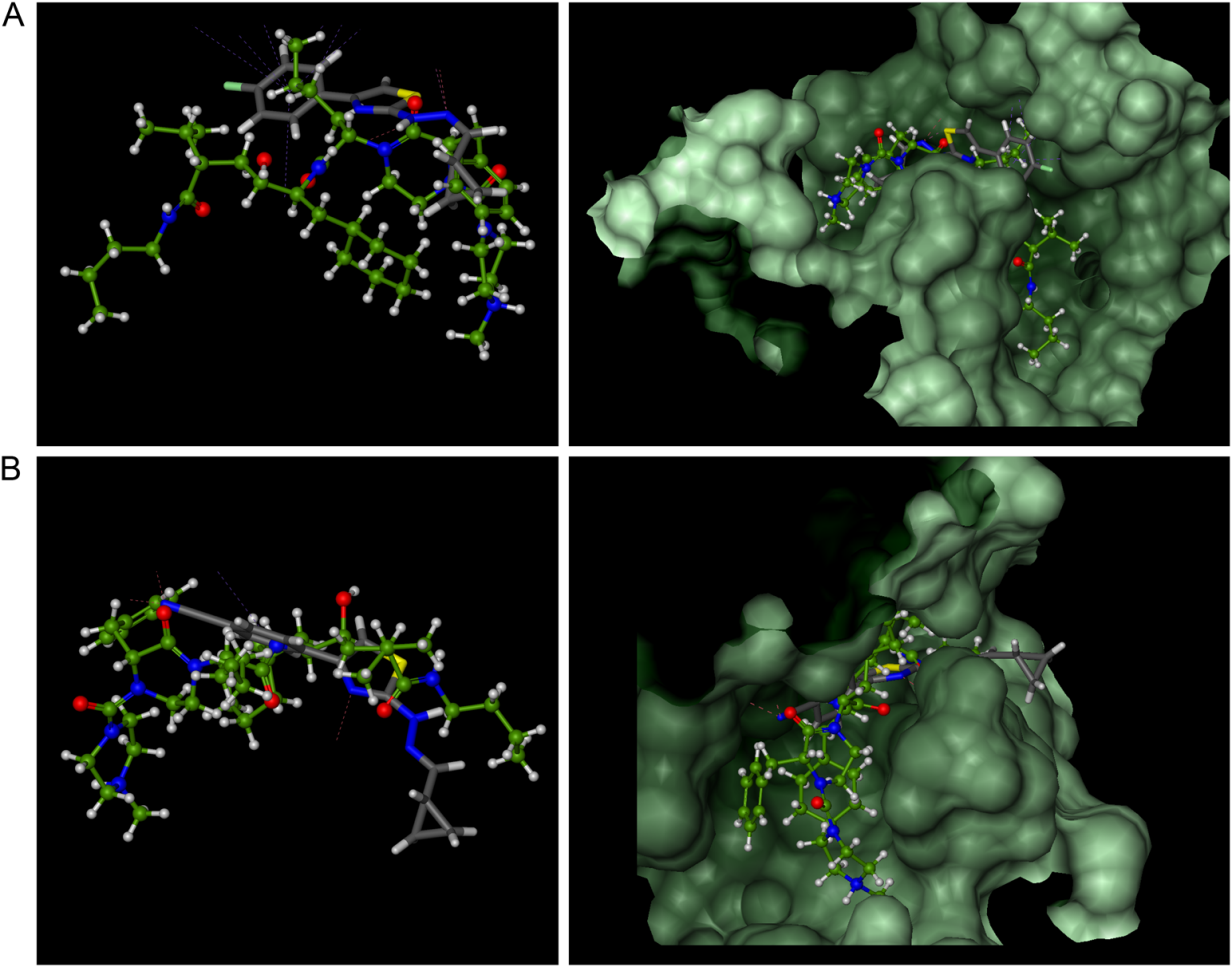
Overlay of calculated binding modes of (in gray): **3a** **a** and **3h** **b** and native ligand A70450 (in green) in the SAP-binding pocket. Remaining compounds are predicted to bind in similar manner

In order to identify potential molecular targets responsible for antifungal and anticonvulsant activity, the molecular docking was performed. We have earlier demonstrated that N-myristoyltransferase (NMT) and secreted aspartic proteinase (SAP) are potential molecular targets for hydrazinylthiazoles. NMT catalyses the reaction of myristate with important fungal cell proteins (Łączkowski et al. 2014b, 2016e). In contrast, SAP is responsible for the adherence of fungal cells to host epithelial cells followed by colonization of the host tissue. Continuing our research on potential inhibitors of these enzymes, we decided to use them in our *in silico* experiments. According to the docking scores presented in (Table 7), in contrast to docking results in NMT-binding site, all compounds **3a**–**3d** and **3f**–**3i** are identified to be binding to the SAP-binding pocket with affinity only slightly lower than those predicted for native inhibitor A70450. The overall binding mode of all of them in their extended conformations is very similar to that of A70450, with the exception of **3h** and **3i** for which a slight shift of their cyclopropane ring outside of the SAP pocket is predicted (Fig. 3).

**Table 7:** Scores of top poses of **3a**–**3d** and **3f**–**3i** docked to proteins: SAP and NMT

Molecular targets			Docking scores (kcal/mol)							Native ligand
	3a	3b	3c	3d	3f	3g	3h	3i	3j	
SAP	−18.0	−15.9	−15.6	−15.9	−13.6	−16.5	−17.8	−20.3	−15.7	−20.4
NMT	−19.8	−18.7	−16.9	−17.5	−18.3	−19.1	−17.4	−24.1	−17.3	−36.3

The binding positions of **3a**–**3d** and **3f**–**3i** are stabilized by rich network of intermolecular interactions with surrounding residues and structural water molecules, and most of these interactions are identical to those observed for crystal structure of A70450 in complex with SAP. The binding of the compounds **3f** and **3i** was also studied by docking in the transient receptor potential vanilloid 6 (TRPV6) which represent highly calcium-selective ion channel (Saotome et al. 2016). Studies have shown a wide distribution of TRPV6 in the brain, which indicates its broad role in the nervous system functions (Kumar et al. 2017). Both compounds are predicted to bind with higher affinity (docking scores −19.8 kcal/mol) than native inhibitor (−18.3 kcal/mol) and fenytoine (−16.4 kcal/mol). Their thiazole ring is positioned close to carboxylic group of native 6-(5-methyl-2-oxo-imidazolidin-4-yl)hexanoic acid while the cyclopropane-hydrazine arms are pulled out of the binding pocket. The calculated binding mode of **3f** and **3i** shows two hydrogen bonds between their nitrogen atoms of hydrazine group and surrounding residues while for **3i** additional H-bond interaction of its terminal azide group with Gln118 is predicted (data given in Supporting Information).

## Conclusion

We have developed an efficient method for the synthesis of new thiazole derivatives containing a cyclopropane fragment. Our results indicated that newly synthesized compounds showed very high antifungal activity towards most reference and clinical strains of *Candida* spp. Their antimicrobial effect was similar and even stronger to nystatin which is a popular antimycotic drug. Additionally, compounds containing chloro and trifluoromethyl substituents showed an interesting anticonvulsant activities in the MES test, whereas compounds containing methoxy and azido substituents demonstrated the anticonvulsant activity in PTZ-induced seizures. Noteworthy, none of these compounds impaired animals’ motor skills in the rotarod test. Moreover, compounds **3a, 3h**, and **3j** showed significant anti-*Toxoplasma gondii* activity, with IC_50_ values 31–52 times lower than those observed for sulfadiazine. The results of the cytotoxicity evaluation, *anti-Candida* spp. and anti-*Toxoplasma gondii* activity studies showed that *Candida* spp. and *Toxoplasma gondii* growth was inhibited at non-cytotoxic concentrations for the host cells. These data showed that new compounds may be used for preclinical investigation in the treatment of candidiasis and microbial infection-related seizures.

## Electronic supplementary material

Supplementary Item
