# Supplementary material for: Thiazoles with cyclopropyl fragment as antifungal, anticonvulsant, and anti-Toxoplasma gondii agents: synthesis, toxicity evaluation, and molecular docking study
Source: Med Chem Res. 2018 Jul 21;27(9):2125–40. doi: 10.1007/s00044-018-2221-x (PMC6133161; doi:10.1007/s00044-018-2221-x)
Supplement: Supplementary file 1 — Supplementary Item [file 44_2018_2221_MOESM1_ESM.doc]

**Supporting Information**

**Thiazoles with cyclopropyl fragment as antifungal, anticonvulsant
and anti-*Toxoplasma gondii* agents. Synthesis, toxicity evaluation, ADME prediction and molecular docking study**

**Krzysztof Z. Łączkowski^1^,*, Natalia Konklewska^1^, Anna Biernasiuk^2^,** **Anna Malm^2^, Kinga Sałat^3^,
Anna Furgała^3^, Katarzyna Dzitko^4^, Adrian Bekier^4^, Angelika Baranowska-Łączkowska^5^, Agata Paneth^6^**

^1^Department of Chemical Technology and Pharmaceuticals, Faculty of Pharmacy, Collegium Medicum, Nicolaus Copernicus University, Jurasza 2, 85-089 Bydgoszcz, Poland,

^2^Department of Pharmaceutical Microbiology, Faculty of Pharmacy, Medical University, Chodźki 1, 20-093 Lublin, Poland

^3^Chair of Pharmacodynamics, Faculty of Pharmacy, Jagiellonian University, Medical College, Medyczna 9, 30-688 Krakow, Poland

^4^Department of Immunoparasitology, University of Lodz, Banacha 12/16, 90-237 Lodz, Poland

^5^Institute of Physics, Kazimierz Wielki University, Plac Weyssenhoffa 11, 85-072 Bydgoszcz, Poland

^6^Department of Organic Chemistry, Faculty of Pharmacy, Medical University of Lublin, Chodźki 4a, 20-093 Lublin, Poland

*Corresponding author: Krzysztof Z. Łączkowski, Department of Chemical Technology and Pharmaceuticals, Faculty of Pharmacy, Collegium Medicum, Nicolaus Copernicus University, Jurasza 2, 85-089 Bydgoszcz, Poland, Tel.: +48 52 5853935; fax: +48 52 585 3920. *E-mail address*: krzysztof.laczkowski@cm.umk.pl

**Contents**

**S1.** Proliferation [%] of *T. gondii* RH strain in the concentration range 1-2500 μg/ml of sulfadiazine ± SD, and the IC_50_ *anti-Tg*.

**S2**. The calculated binding modes of **3f** (A), **3i** (B) and native ligand 6-(5-methyl-2-oxo-imidazolidin-4-yl)hexanoic acid (in green) in the calcium channel receptor (PDB entry: 5IWP).

**S3**. Estimation of IC_50_ [μg/ml] values of **3a**, **3h** and **3j** compounds in *T. gondii* RH strain infected VERO cells.

**S4.** Representative ^1^H, ^13^C NMR and GC-EI-MS spectra for compounds **2**, **3a**, **3d** and **3i**.

**S1**

| **Sulfadiazine** | | | | | | | | | | |
| --- | --- | --- | --- | --- | --- | --- | --- | --- | --- | --- |
| **Concentration [µg/ml]** | **2500** | **1250** | **500** | **250** | **125** | **50** | **25** | **5** | **1** | **IC_50_ [µg/ml]** |
| Proliferation of *Tg* [%] | *45.36 ±0.58* | *45.17 ±1.03* | *56.68 ±3.64* | *61.79 ±3.13* | *65.58 ±4.80* | *67.99 ±7.73* | *78.58 ±4.67* | *96.71 ±3.85* | *104.59 ±4.12* | **935.8** |

**S2**

| (A) |  | (B) |
| --- | --- | --- |
| 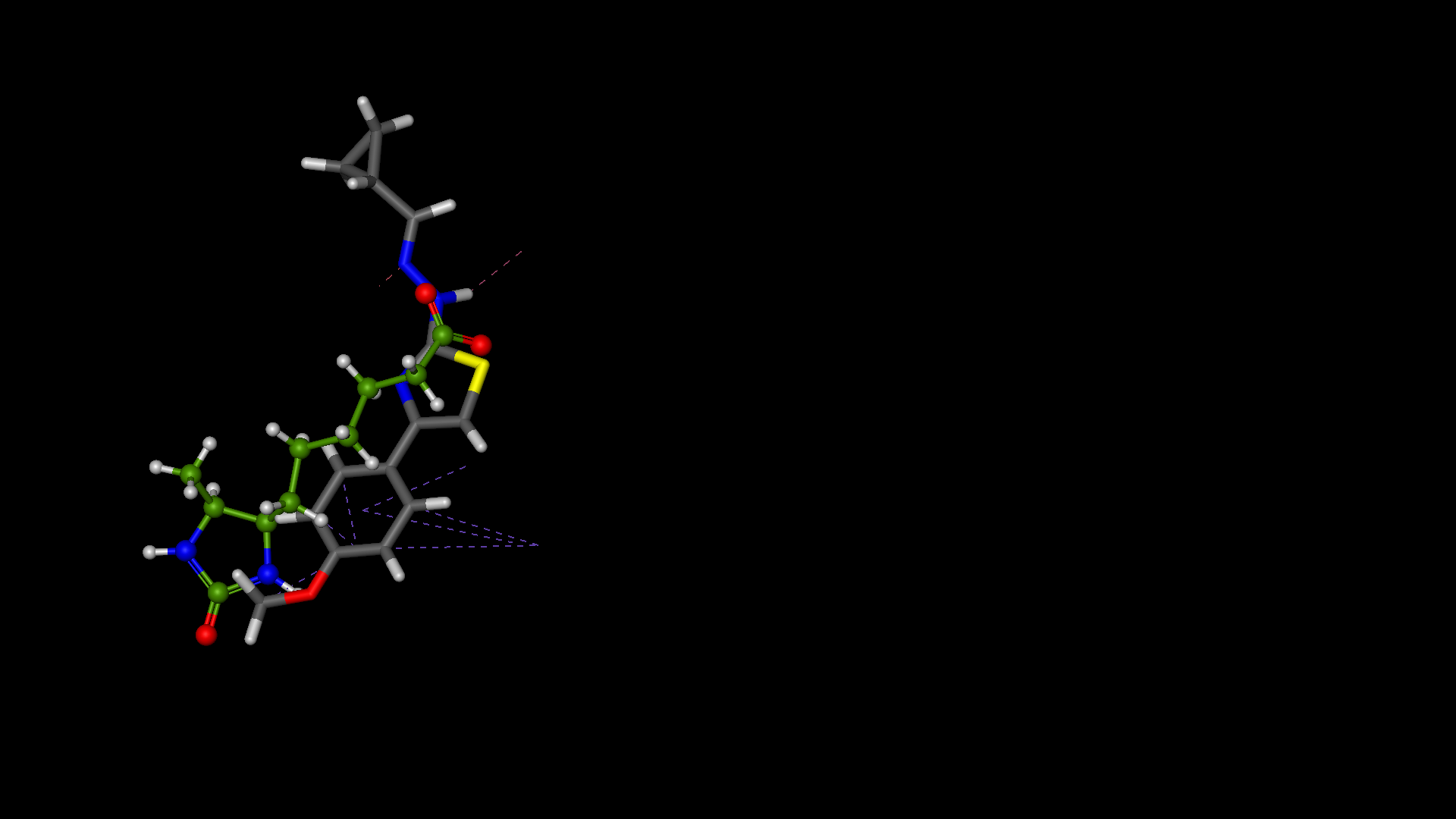 |  | 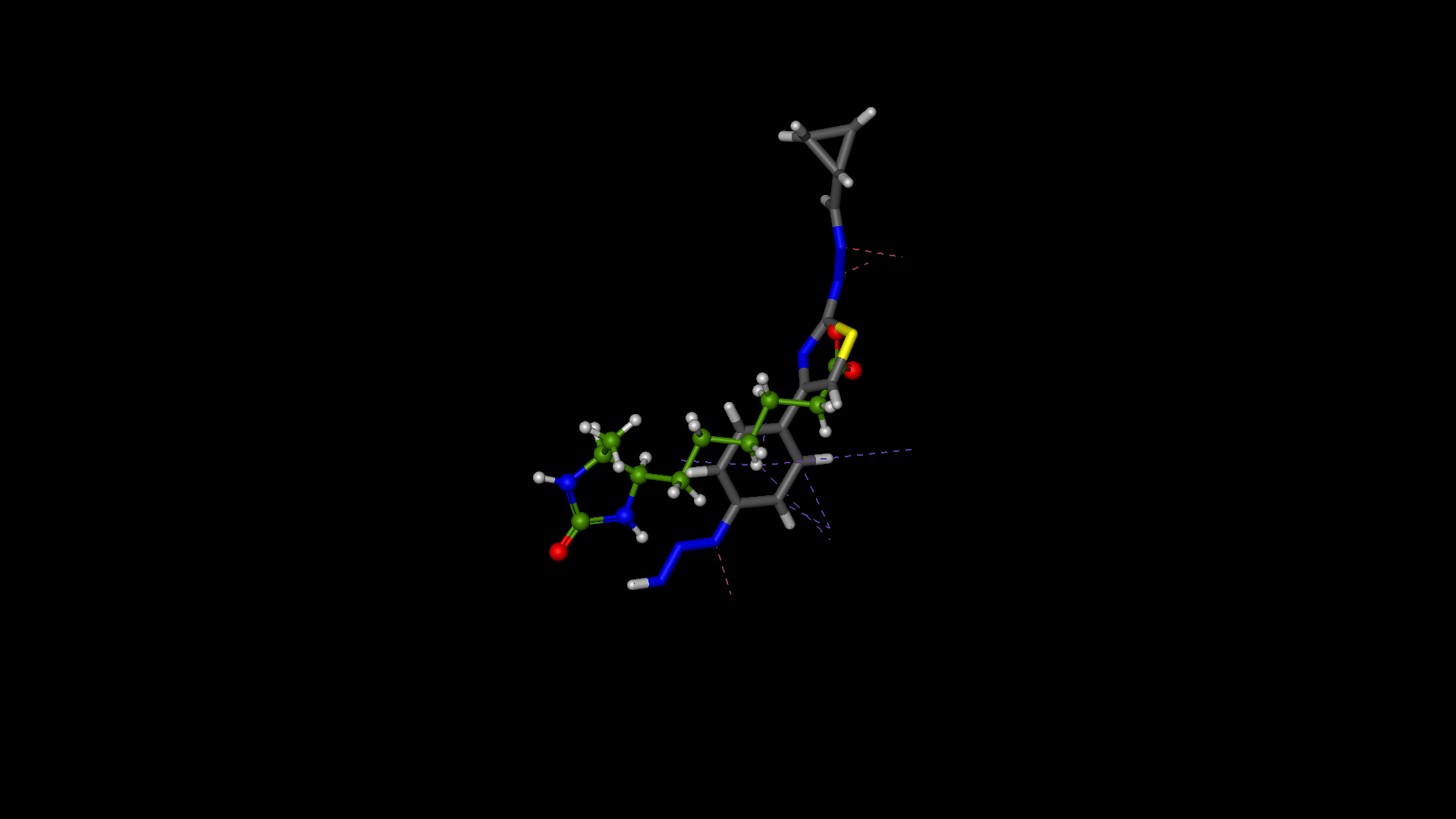 |
|  |  |  |
| 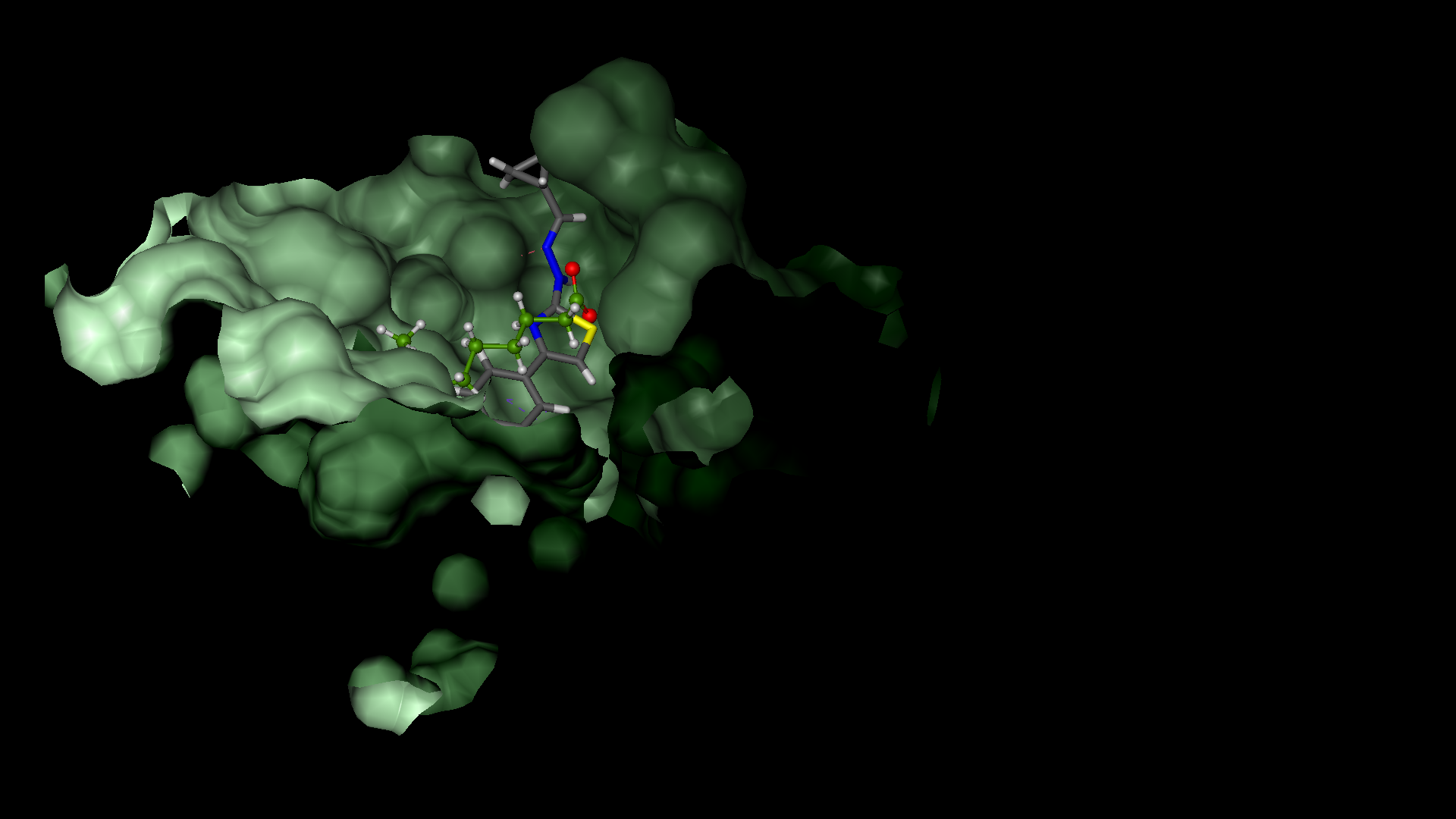 |  | 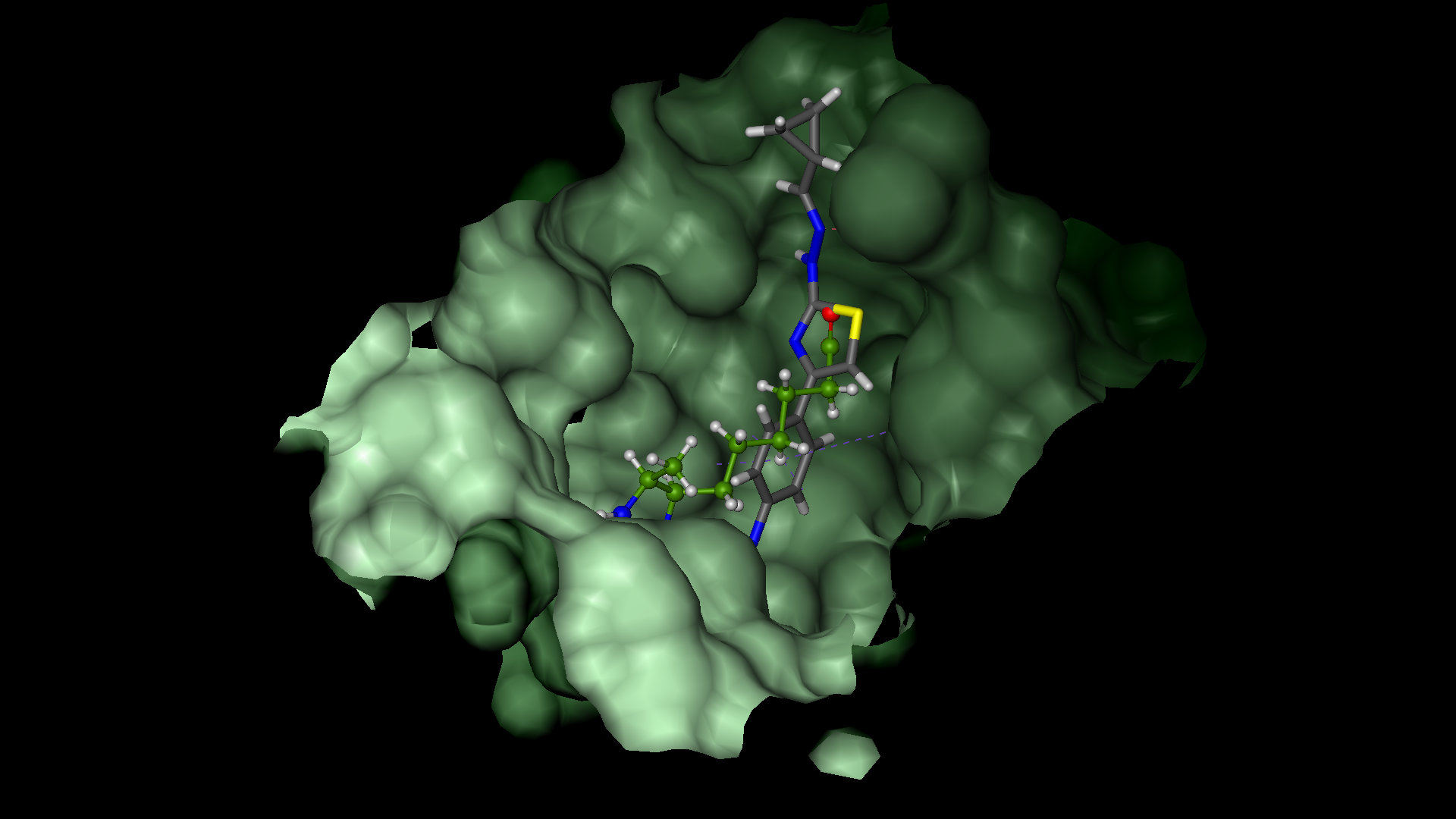 |
| 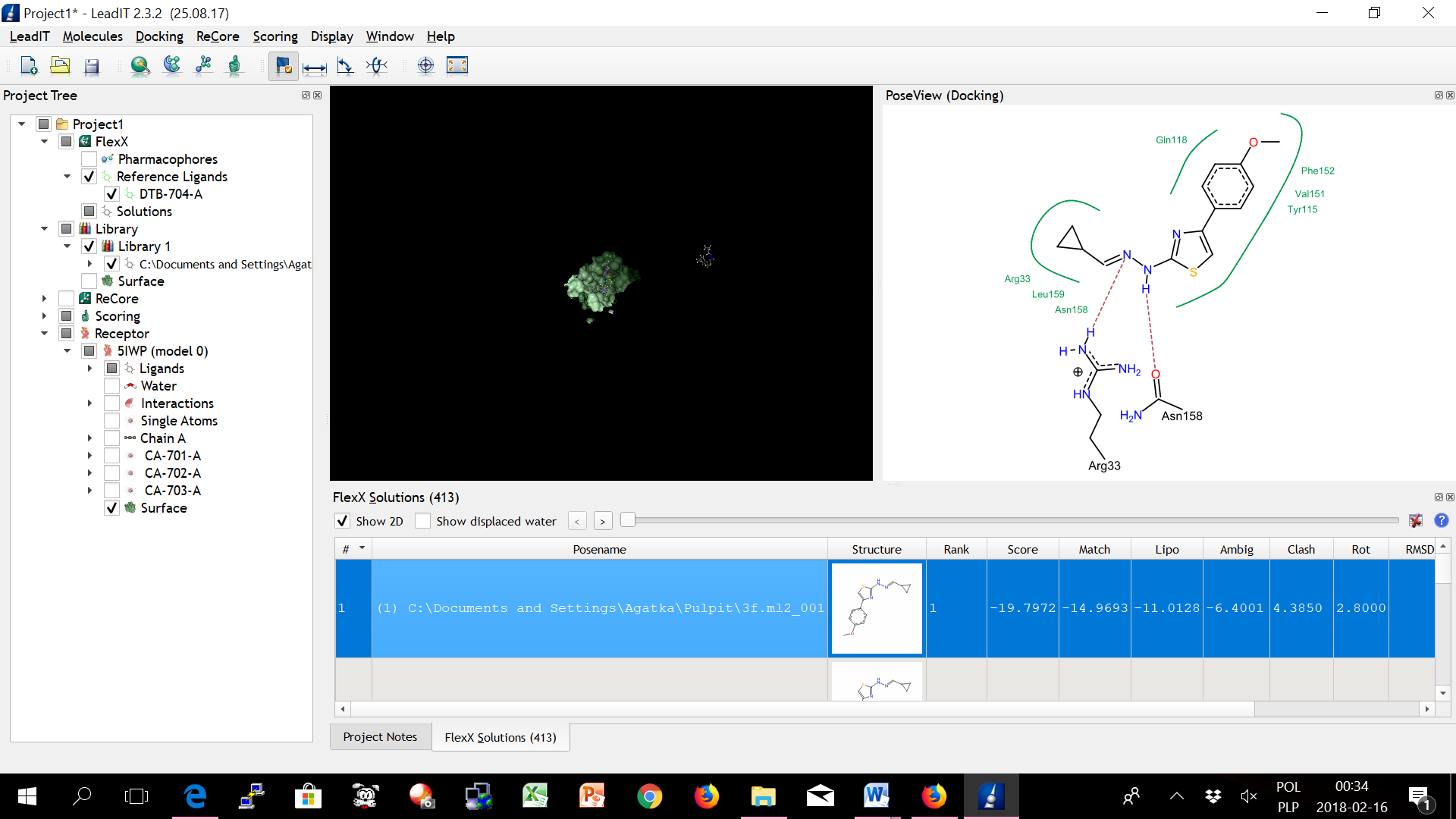 |  | 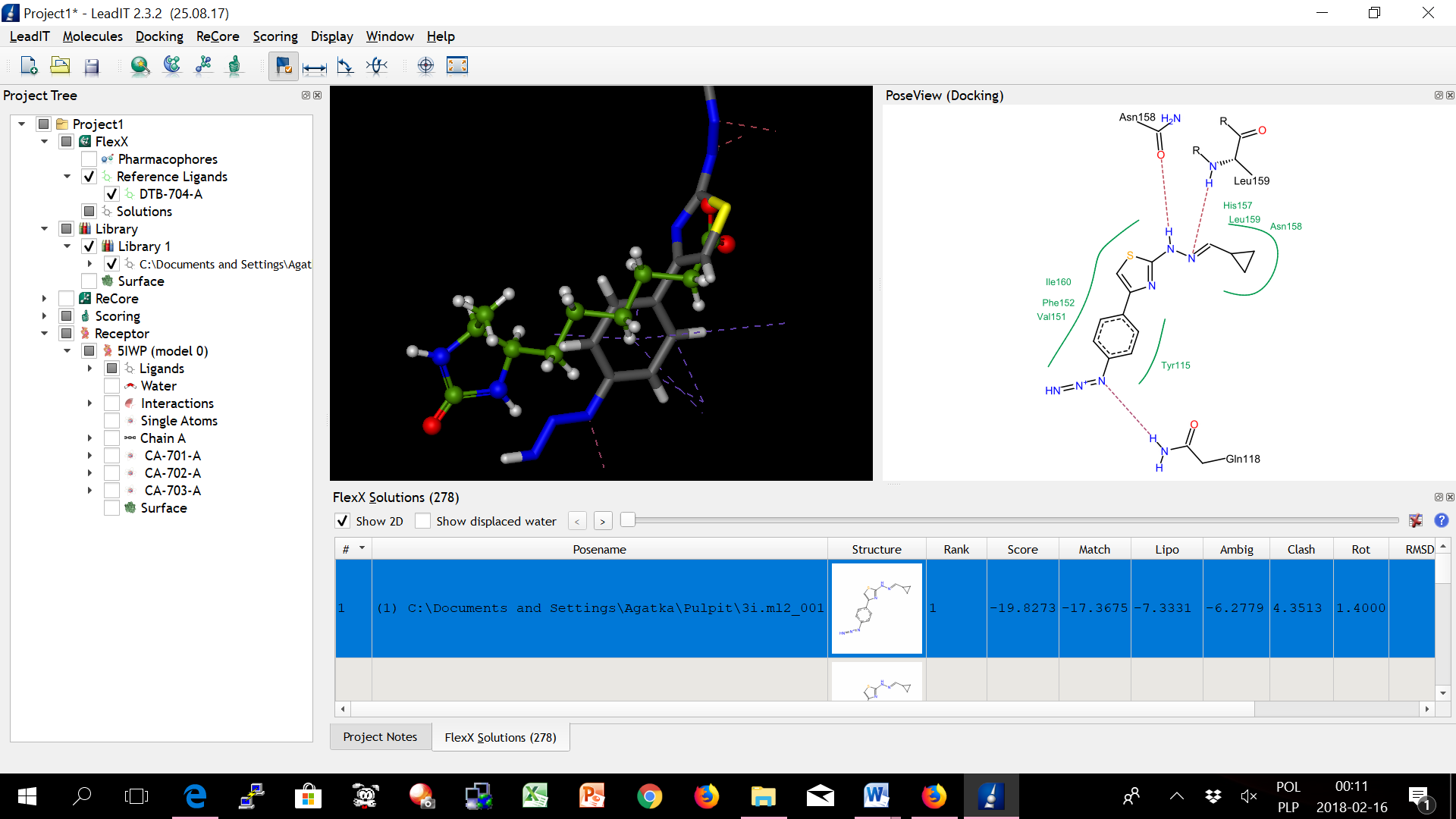 |

**S3**

**

**

**S4**
